# Development of Lentiviral Vectors for HIV-1 Gene Therapy with Vif-Resistant *APOBEC3G*

**DOI:** 10.1016/j.omtn.2019.10.024

**Published:** 2019-10-31

**Authors:** Krista A. Delviks-Frankenberry, Daniel Ackerman, Nina D. Timberlake, Maria Hamscher, Olga A. Nikolaitchik, Wei-Shau Hu, Bruce E. Torbett, Vinay K. Pathak

**Affiliations:** 1 Viral Mutation Section, HIV Dynamics and Replication Program, National Cancer Institute at Frederick, Frederick, MD 21702, USA; 2 The Scripps Research Institute, La Jolla, CA 92073, USA; 3 Viral Recombination Section, HIV Dynamics and Replication Program, National Cancer Institute at Frederick, Frederick, MD 21702, USA

**Keywords:** APOBEC3G, HIV-1, lentiviral vector, gene therapy, self-activating vector, D128K, hypermutation, CD34+ HSPC, CD4+ T cells

## Abstract

Strategies to control HIV-1 replication without antiviral therapy are needed to achieve a functional cure. To exploit the innate antiviral function of restriction factor cytidine deaminase *APOBEC3G* (*A3G*), we developed self-activating lentiviral vectors that efficiently deliver HIV-1 Vif-resistant mutant *A3G-D128K* to target cells. To circumvent APOBEC3 expression in virus-producing cells, which diminishes virus infectivity, a vector containing two overlapping fragments of *A3G-D128K* was designed that maintained the gene in an inactive form in the virus-producer cells. However, during transduction of target cells, retroviral recombination between the direct repeats reconstituted an active *A3G-D128K* in 89%–98% of transduced cells. Lentiviral vectors that expressed A3G-D128K transduced CD34^+^ hematopoietic stem and progenitor cells with a high efficiency (>30%). A3G-D128K expression in T cell lines CEM, CEMSS, and PM1 potently inhibited spreading infection of several HIV-1 subtypes by C-to-U deamination leading to lethal G-to-A hypermutation and inhibition of reverse transcription. SIVmac239 and HIV-2 were not inhibited, since their Vifs degraded A3G-D128K. A3G-D128K expression in CEM cells potently suppressed HIV-1 replication for >3.5 months without detectable resistant virus, suggesting a high genetic barrier for the emergence of A3G-D128K resistance. Because of this, *A3G-D128K* expression in HIV-1 target cells is a potential anti-HIV gene therapy approach that could be combined with other therapies for the treatment and functional cure of HIV-1 infection.

## Graphical Abstract

**Figure:**
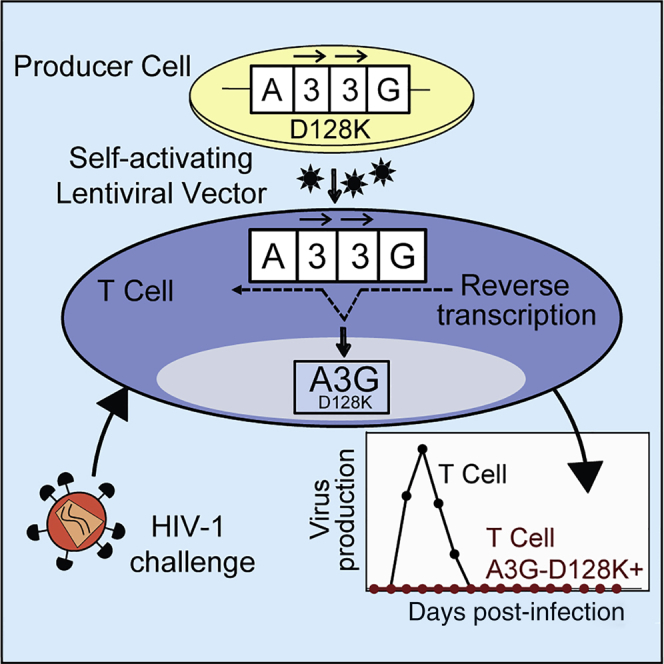

## Introduction

Treatment for HIV-1 infection with potent combination antiretroviral therapy (cART) can control viral replication and delay onset of AIDS.^1^ However, long-lived latently infected cells can persist and rekindle viral infection upon therapy interruption, resulting in lifelong cART associated with high costs, toxicities, incomplete adherence, and drug resistance. Therefore, strategies that can eradicate most infected cells from patients or control viral replication in the absence of cART to achieve a functional cure are needed.^2^ The apparent cure of the “Berlin patient” and recent long-term HIV-1 remission of another patient by transplantation of hematopoietic stem cells that did not express coreceptor chemokine receptor 5, or CCR5, have raised hopes of a cure.^3^^,^^4^ Unfortunately, other cases of allogeneic stem cell transplantation have led to viral rebound^5^, ^6^, ^7^ and/or the emergence of rare pre-existing C-X-C chemokine receptor type 4, or CXCR4 variants,^8^ suggesting that novel strategies are needed to achieve a functional cure for HIV-1 infection.

One potential approach to controlling HIV-1 replication without cART is to interfere with HIV-1’s ability to counteract innate host restriction factors, such as the APOBEC3 family of cytidine deaminases,^9^ the tripartite interaction motif 5 alpha protein (TRIM5α),^10^^,^^11^ the sterile alpha motif (SAM) domain and histidine-aspartate (HD) domain-containing protein 1 (SAMHD1),^12^^,^^13^ the BST2/tetherin protein,^14^^,^^15^ and the MXB/MX2 protein.^16^, ^17^, ^18^ Restriction factor APOBEC3G (A3G)^9^ is a cellular cytidine deaminase that is packaged into newly formed virions and, upon subsequent infection, induces C-to-U deamination of the minus-strand DNA during reverse transcription, resulting in lethal G-to-A hypermutations.^19^^,^^20^ A3G can also restrict HIV-1 through deaminase-independent mechanisms, leading to the inhibition of reverse transcription^21^, ^22^, ^23^ and integration.^22^^,^^24^^,^^25^ HIV-1 has evolved to combat A3G by expressing Vif, which targets A3G for polyubiquitination and proteasomal degradation,^26^^,^^27^ thereby preventing its virion incorporation.

Therapeutic strategies based on expressing a Vif-resistant A3G mutant in HIV-1 target cells is an attractive approach to HIV-1 gene therapy,^26^ but efficient delivery of a functional *A3G* to target cells is technically challenging. Viral vectors will inevitably express A3G in the producer cells, and its incorporation into virions will inactivate the vector and the vector-encoded *A3G*. Previous gene therapy studies have proposed to utilize A3G to block HIV-1 replication by inhibiting A3G ubiquitination,^28^ increasing virion incorporation of Vif-resistant A3G mutants,^29^^,^^30^ expressing an HIV-1 Vif-resistant African green monkey A3G,^31^ and using an inducible promoter to block expression of A3G in the producer cells.^32^ Each of these studies tried to avert the potent inhibitory effects of A3G in the virus-producing cells but faced difficulties in achieving efficient delivery of *A3G* to target cells. Ao et al.^29^^,^^33^ used an adeno-associated viral vector system (AAV2/5) to introduce Vif-resistant mutant *A3G-P129A* into peripheral blood mononuclear cells (PBMCs) and macrophages, but potent inhibition of HIV-1 replication was not observed in PBMCs, perhaps because AAV2/5 did not efficiently transduce human CD4^+^ T cells. Voit et al.^34^ inserted *A3G-D128K* along with a dominant-negative mutant of HIV-1 Rev (RevM10) and human/rhesus TRIM5α by gene editing into the *CCR5* locus. Although the efficiency of gene delivery was not addressed, expression of A3G-D128K alone was shown to provide the strongest protection (100- to 200-fold) from HIV-1 replication compared to human/rhesus TRIM5α and RevM10. All these approaches have been hampered by low efficiency of transduction, inability to transduce the natural target cells of HIV-1 infection, and low efficiency of genome editing.

We previously described the development of self-activating retroviral vectors for gene therapy using directly repeated nucleotide sequences.^35^^,^^36^ As outlined by the dynamic copy choice model for retroviral recombination,^37^ duplicated gene sequences are precisely and accurately deleted at a high efficiency by homologous recombination during the process of reverse transcription.^35^^,^^38^, ^39^, ^40^, ^41^, ^42^, ^43^ Here, we developed a self-activating lentiviral vector that utilized directly repeated sequences of a Vif-resistant mutant of *A3G* (*A3G-D128K*) to prevent expression of A3G-D128K in the lentivector producer cells but reconstituted a functional *A3G-D128K* in the target cells during retroviral transduction. The results demonstrate that the vectors can be used to efficiently transduce CD4^+^ T cell lines and hematopoietic stem and progenitor cells (HSPCs). Importantly, the *in vitro* experiments indicated that selection for A3G-D128K-resistant HIV-1 variants has a high genetic barrier, adding further support to potential anti-HIV gene therapy by the expression of A3G-D128K to control HIV-1 replication and spread.

## Results

### Self-Activating Lentiviral Vectors for Efficient Delivery of *A3G-D128K*

**Figure 1:**
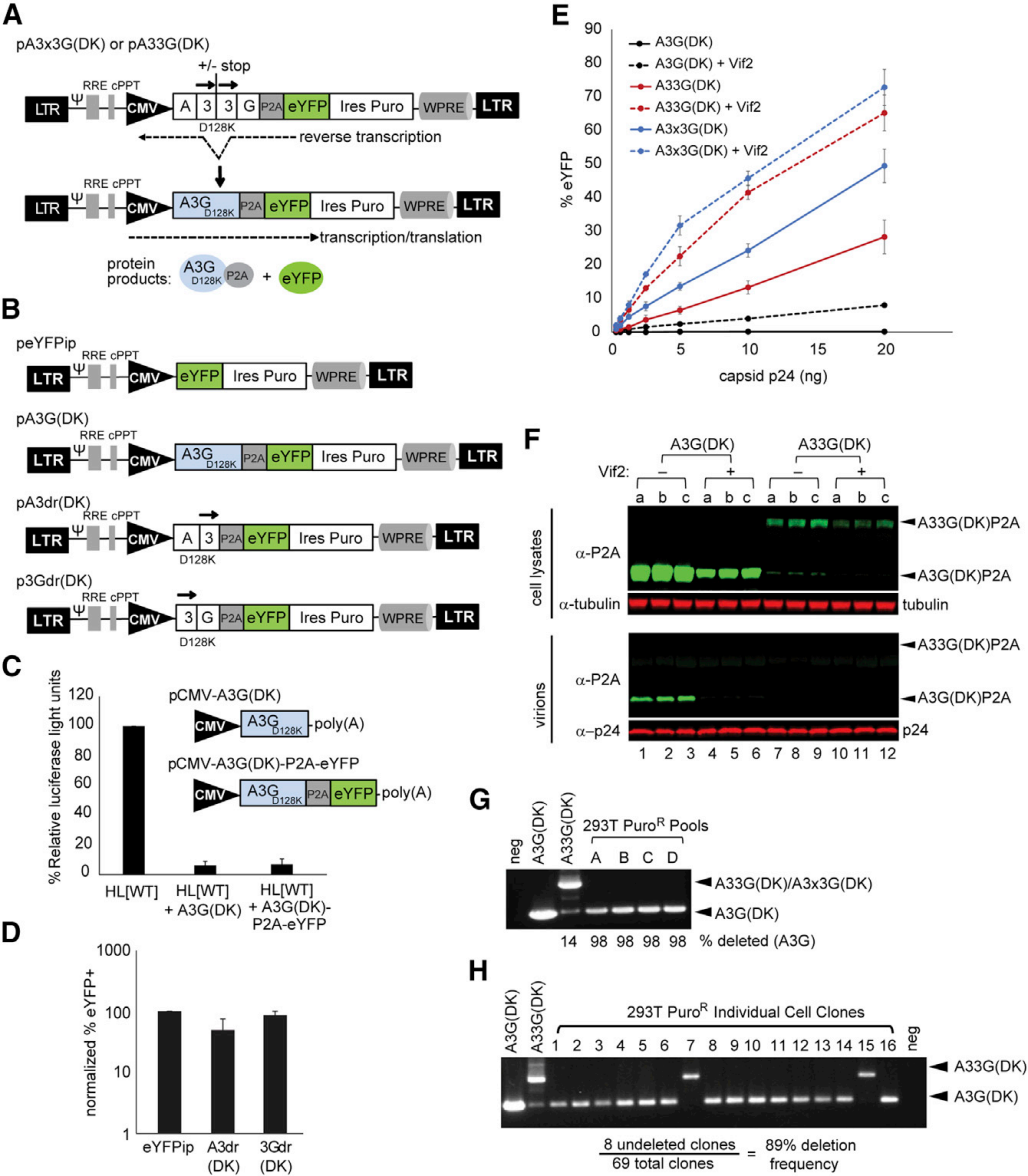
A3G-D128K Direct Repeat Vectors, Transduction, and Frequency of A3G Reconstitution (A) Lentiviral vectors pA3x3G(DK) and pA33G(DK) contain an overlapping ~900-bp homologous region of *A3G-D128K* (“3,” indicated by black arrows). *A3* and *3G* are the 5′ and 3′ fragments of *A3G-D128K*, respectively, separated by three stop codons (+stop; “x”) or no stop codons (-stop), fused in frame with the self-cleaving porcine teschovirus-1 2A peptide (P2A) and enhanced yellow fluorescent protein (*eYFP*). An internal promoter from cytomegalovirus (CMV), an internal ribosomal entry site (IRES), and a puromycin (*puro*)-selectable marker are present, followed by the woodchuck hepatitis virus post-transcriptional regulatory element (WPRE). These vectors contain intact long terminal repeats (LTRs). Indicated below the vector are the protein products that are expected following viral infection, direct repeat deletion during reverse transcription, translation, and P2A cleavage. RRE, Rev response element; Ψ, HIV-1 RNA packaging signal; cPPT, central polypurine tract. (B) Vector peYFPip expresses *eYFP*, and vector pA3G(DK) expresses the intact A3G-D128K fused in frame with P2A and eYFP. Vectors pA3dr(DK) and p3Gdr(DK) express the *A3* and *3G* portions of the *A3G-D128K* direct repeat, respectively. (C) Normalized p24 capsid protein (CA) from HL[WT] virus made in the presence of CMV-driven vectors expressing A3G-D128K or A3G-D128K-P2A-eYFP was used to transduce 293T target cells. Indicated are the average luciferase light units normalized to HL[WT] in the absence of A3G-D128K (100%) (n = 3). (D) Normalized p24 CA from each vector virus was used to transduce 293T target cells; indicated is the percentage of eYFP^+^ cells 48 h post-transduction normalized to eYFPip (100%) (n = 3). (E) Normalized p24 CA from vector virus pA3G(DK), pA33G(DK), and pA3x3G(DK) produced in the presence or absence of Vif2 was used to transduce 293T target cells, and the proportion of eYFP^+^ cells 48-h post-transduction was determined. (F) Western blot analysis of cell lysates and virions showing expression and encapsidation of A3G(DK)P2A and/or A33G(DK)P2A. (G) Puromycin-resistant pools of 293T cells transduced with A3x3G(DK) virus were analyzed for the deletion frequency of the A3G-D128K ~900-bp direct repeat. Primers flanking A3G(DK) were used for PCR amplification, with positive plasmid controls indicated for A3G(DK) (2,190 bp) and undeleted A33G(DK) (1,290 bp). Band density analysis indicates an average 98% direct repeat deletion frequency for 4 independent pools (A–D). Low levels of deletion were detected with the plasmid A33G(DK) control due to direct repeat deletion during PCR (14%). (H) Puro selected 293T cell clones transduced with A33G(DK) virus. Primers flanking A3G(DK) were used for PCR amplification with plasmid controls shown for A3G(DK) (1,864 bp) and undeleted A33G(DK) (964 bp). PCR analysis shows an average 89% direct repeat deletion frequency from three independent transductions (69 total clones). Error bars represent SD.

Human CD4^+^ T cells express A3G, which, in the absence of the virally encoded Vif, potently inhibits HIV-1 replication. A3G mutant D128K (A3G-D128K) is resistant to HIV-1 Vif-mediated degradation, and its expression in human CD4^+^ T cells should result in potent inhibition of wild-type HIV-1 replication and spread. Lentiviral vectors are one of the most efficient vehicles for delivery of therapeutic genes to human CD4^+^ T cells. However, delivery of *A3G-D128K* using a traditional lentiviral vector is inefficient, since expression of A3G-D128K in the virus-producing cells results in its virion incorporation, leading to drastic loss of virion infectivity and lethal hypermutation of the therapeutic gene. To prevent expression of A3G-D128K in the lentivector producer cells, we constructed lentiviral vectors that encoded two overlapping fragments of *A3G-D128K* (referred to as *A3* and *3G*; Figure 1A). One copy of the overlapping ∼900-bp direct repeat is efficiently deleted during reverse transcription by HIV-1 reverse transcriptase switching templates in the homologous repeats, resulting in the functional reconstitution of *A3G-D128K* (Figure S1; reviewed by Delviks-Frankenberry et al.^35^). Briefly, during RNA-dependent DNA synthesis of the 3′ direct repeat (the “3” portion of 3G), the RNase H activity of reverse transcriptase degrades the template RNA, which allows the nascent DNA strand to anneal to the cRNA in the 5′ direct repeat (the “3” portion of A3). Subsequently, the reverse transcriptase and the growing point of the nascent DNA dissociate from the 3′ direct repeat and anneal to the 5′ direct repeat, resulting in the deletion of one copy of the direct repeat and any intervening sequences. Because the A3 and 3G portions of A3G do not express a catalytically active A3G, functional A3G-D128K is not expressed in the virus-producing cells and, therefore, cannot inhibit virion infectivity, but a reconstituted *A3G-D128K* is expressed in the target cells, leading to inhibition of subsequent rounds of HIV-1 replication.

Vectors pA33G(DK) and pA3x3G(DK) were constructed with and without stop codons between *A3* and *3G* and fused with a self-cleaving porcine teschovirus-1 2A peptide (P2A)^44^ and enhanced yellow fluorescent protein (*eYFP*); the vectors also expressed the puromycin-resistance gene (*puro*) from an internal ribosomal entry site (IRES) (Figure 1A). Control vectors were also constructed that expressed eYFP (peYFPip), A3G-D128K (pA3G(DK)), and the *A3* (pA3dr(DK)) or the *3G* portions (p3Gdr(DK)) of *A3G-D128K* (Figure 1B).

Control vector pCMV-A3G(DK) expressed an untagged A3G-D128K from a cytomegalovirus (CMV) promoter, whereas the pCMV-A3G(DK)-P2A-eYFP vector expressed A3G-D128K-P2A fusion protein and eYFP after cleavage between P2A and eYFP (Figure 1C). To compare the antiviral activity of A3G-D128K-P2A with A3G-D128K, virus was prepared from cells that expressed A3G-D128K, A3G-D128K-P2A fusion protein, or no A3G in the presence of the HIV-1 luciferase vector, pHL[WT]. Relative luciferase activities in transduced cells showed that expression of A3G-D128K or A3G-D128K-P2A potently inhibited lentivector HL[WT] transduction to 6% and 7%, respectively, compared to the lentivector prepared in the absence of A3G (Figure 1C), indicating that the presence of the P2A tag did not significantly affect the antiviral activity of A3G-D128K.

To test whether the *A3* and *3G* portions of A3G-D128K retained any antiviral activity, virions produced from eYFPip, A3dr(DK), and 3Gdr(DK) vectors were normalized for p24 capsid protein (CA) amounts and used to transduce 293T cells (Figure 1D). Flow cytometry analysis of the transduced cells showed that expression of the A3 (A3dr) or 3G (3Gdr) truncated proteins of A3G-D128K in the lentivector producer cells resulted in <2-fold inhibition of transduction (∼50% and ∼87% eYFP^+^ cells compared to cells transduced with eYFPip, respectively). Expression of A3dr resulted in ∼50% inhibition of transduction, suggesting that A3dr may be packaged into the virions and suppress lentivector transduction by inhibition of DNA synthesis. In future studies, it may be possible to shorten the A3 portion and shift the ∼900-bp direct repeat to suppress the ∼2-fold inhibition of lentivector transduction.

### Self-Activating Vectors Increase the Efficiency of A3G-D128K Transduction

A3G-D128K transduction efficiency was determined for vectors with (A3x3G(DK)) and without (A33G(DK)) stop codons between the direct repeats by transducing 293T cells with serial dilutions of viruses (Figure 1E). Recombination between the repeated regions during transfection of the virus-producing cells could potentially reconstitute *A3G-D128K*; therefore, viruses were prepared in the presence or absence of HIV-2 Vif (Vif2), which can induce degradation of A3G-D128K.^45^ The results showed that, in the absence of Vif2 co-expression, the transduction efficiency with the A3G(DK) vector was nearly undetectable, while the transduction efficiency with the A33G(DK) and A3x3G(DK) viruses was increased to 28% and 49%, respectively (compare cells transduced with 20 ng p24 CA-containing virus). Co-expression of Vif2 increased the transduction efficiencies of the A3G(DK), A33G(DK), and A3x3G(DK) viruses to 8%, 65%, and 73%, respectively. The results indicated that the stop codons between the *A3* and *3G* direct repeats in the A3x3G(DK) vector prevented expression of a A33G-D128K fusion protein that retained antiviral activity and resulted in the highest amounts of infectious lentivectors that expressed eYFP in the target cells.

Next, we analyzed the expression of A3G-D128K and A33G-D128K in the lentivector producer cells and their incorporation into virions by western blotting (Figure 1F). A3G-D128K-P2A and A33G-D128K-P2A fusion proteins were readily detected in the absence of Vif2 (lanes 1–3 and 7–9, respectively) or the presence of Vif2 (lanes 4–6 and 10–12, respectively). A3G-D128K-P2A was also detected at a very low level in cells transfected with A33G(DK) in the absence of Vif2 (lanes 7–9) but not in the presence of Vif2 (lanes 10–12), confirming that *A3G-D128K* was reconstituted at a low efficiency by DNA recombination during transfection and that the A33G-D128K fusion protein was targeted for degradation by Vif2. Virion incorporation of A33G-D128K-P2A or the reconstituted A3G-D128K-P2A could not be detected because of low levels of virion incorporation and sensitivity of detection. A3G-D128K-P2A was efficiently packaged into virions, and co-expression of Vif2 substantially reduced virion incorporation of A3G-D128K but did not eliminate it, since a faint band was detectable upon overexposure of the blot (data not shown).

To determine the frequency of *A3G-D128K* reconstitution during reverse transcription, 4 independent pools of 293T cells transduced with the A3x3G(DK) virus were generated, and their genomic DNA was used to PCR-amplify proviral DNA using primers flanking the A3x3G sequence (Figure 1G). The results showed that most of the amplified DNA (∼98%) generated a 1,290-bp band labeled A3G(DK) that was expected from direct repeat deletion, while a 2,190-bp band that was expected in the absence of deletion was undetectable. PCR amplification of the A33G(DK) vector DNA indicated that direct repeat deletion occurred in ∼14% of the amplified DNA fragments, indicating that most of the direct repeat deletion occurred during reverse transcription and not during PCR amplification. To determine the frequency of *A3G-D128K* gene reconstitution more precisely, 69 single-cell clones of transduced cells that were selected for puromycin resistance were isolated and analyzed by PCR amplification (Figure 1H). *A3G-D128K* reconstitution was observed in 61 of the 69 cell clones, providing a reconstitution frequency of 89%, which was similar to the previously observed >87% deletion frequency for a 971-bp repeated sequence.^38^

### Self-Activating A3x3G-D128K-Expressing Vector Reduces Hypermutation and Viral Inactivation

We determined the extent to which proviral DNAs in target cells transduced with the A3G(DK), A33G(DK), and A3x3G(DK) vectors were hypermutated by PCR amplification and sequencing of a 982-bp proviral DNA fragment (Figure S2; Table 1). In the absence of Vif2 co-expression, a high frequency of A3G(DK) proviral DNAs was hypermutated (52/52 clones), A33G(DK) proviral DNAs were hypermutated to a lesser extent (28/55 clones), and very few A3x3G(DK) proviral DNAs were hypermutated (3/63 clones). Interestingly, the higher frequency of hypermutation observed for the A33G(DK) proviral DNAs compared to the A3x3G(DK) vector suggested that the A33G-D128K fusion protein was expressed, incorporated into virions, and retained cytidine deaminase activity. Co-expression of Vif2 resulted in lower hypermutation frequencies for all three vectors, although a high frequency of hypermutation was still observed for A3G(DK) proviral genomes (40/60 clones). Proviral genomes derived from the A3x3G(DK) vector had ∼65-fold and ∼164-fold fewer G-to-A mutations than the proviral genomes from the A3G(DK) vector in the absence or presence of Vif2, respectively (Table 1). Overall, the A3x3G(DK) self-activating vector, in the presence of Vif2 co-expression, efficiently suppressed A3G-D128K expression in the lentivector producer cells and inactivation of the viral vector by hypermutation.

**Table 1:** Frequency of Hypermutated Clones and G-to-A Hypermutation Profile for A3G-D128K, A33G-D128K, and A3x3G-D128K Virus Produced in the Absence or Presence of Vif2

Vector and Presence or Absence of Vif2	No. of Hypermutated Clones/Total Clones Sequenced (%)	Average G-to-A Mutations/Hypermutated Clone	Dinucleotide Context of G-to-A Mutations				Total G-to-A Mutations	Total G-to-A Mutations/Total Clones Sequenced
			GG	GA	GC	GT		
**A3G(DK)**								

−	52/52 (100%)	40.3	1,851	227	47	86	2,116	40.7
+	40/60 (66.7%)	18.5	678	44	6	10	738	12.3

**A33G(DK)**								

−	28/55 (50.9%)	13.1	319	16	3	8	346	6.3
+	3/84 (3.6%)	15.8	67	8	7	2	84	1

**A3x3G(DK)**								

−	3/63 (4.7%)	11.6	35	1	2	2	40	0.63^a^
+	0/53 (0%)	0	1	1	2	0	4	0.075^a^

a Fold difference in G-to-A mutations for A3G(DK) versus A3x3G(DK) in the absence of Vif2 (40.7/0.63 = 65-fold) or presence of Vif2 (12.3/0.075 = 164-fold).

### Selection of T Cell Populations That Stably Express A3G-D128K

**Figure 2:**
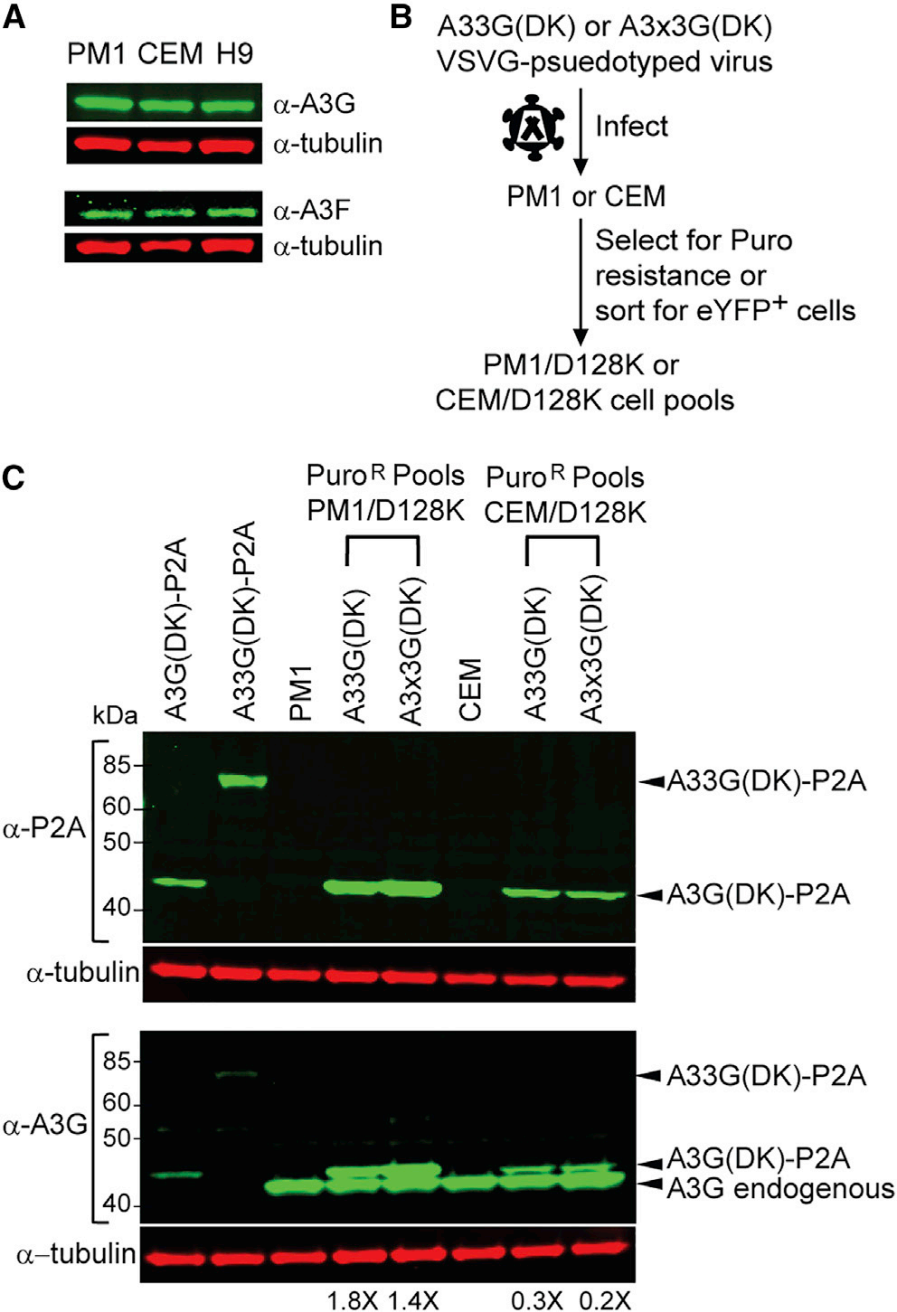
CEM/D128K and PM1/D128K Cell Lines (A) Western blot analysis of endogenous expression levels of A3G and A3F in CEM, PM1, and H9 T cell lines. (B) Protocol used to create A3G-D128K-expressing PM1/D128K and CEM/D128K cell lines. (C) Western blot analysis of puromycin-resistant CEM/D128K and PM1/D128K cell lines (n = 5). Upper blot probed with α-P2A antibody (green bands) and α-tubulin antibody (red bands). Lower blot probed with α-A3G antibody (green bands) and α-tubulin antibody (red bands). Two representative puromycin-resistant A3G-D128K-expressing CEM and PM1 cell pools (derived from A33G(DK) or A3x3G(DK) vector virus) are indicated. Lanes labeled A3G-D128K-P2A and A33G-D128K-P2A show deleted ~46-kDa and undeleted ~82-kDa bands from transfected 293T cells, respectively.

We verified that endogenous A3G and A3F expression in CD4^+^ T cell lines 174XCEM (hereinafter referred to as CEM) and PM1 was similar to that in H9 T cells (Figure 2A); A3G and A3F expression in H9 cells was previously determined to be comparable to that in primary CD4^+^ T cells.^46^, ^47^, ^48^ The CEM cells can be infected with CXCR4-tropic HIV-1 virions, while the PM1 cells can be infected with CXCR4-tropic as well as CCR5-tropic virions. Either CEM and PM1 cells were transduced with the A33G(DK) or A3x3G(DK) lentivectors and placed directly on puromycin selection, or eYFP^+^ cells were first sorted using flow cytometry and then placed on puromycin selection (Figure 2B). Western blotting analysis of puromycin-resistant PM1 (PM1/D128K) or CEM (CEM/D128K) cell pools indicated that *A3G-D128K* reconstitution was efficient and that the A3G-D128K-P2A fusion protein was expressed (Figure 2C). The undeleted A33G(DK)-P2A protein could be detected in 293T cells transfected with this vector using an anti-P2A or an anti-A3G antibody but not in the PM1/D128K and CEM/D128K cell pools. The intensities of the endogenous A3G and the slightly larger A3G(DK)-P2A bands detected using an anti-A3G antibody indicated that the A3G(DK)-P2A protein levels were slightly higher than that for the endogenous A3G protein in PM1 cells (1.4- to 1.8-fold) and were at 20%–30% of the endogenous A3G protein in the CEM cells. Stable A3G-D128K expression in the CEM and PM1 cell lines did not have a detectable effect on their viability or growth kinetics (Figures S3A and S3B).

### A3G-D128K Potently Inhibits Replication of Multiple HIV-1 Subtypes and Subtype-B Patient Isolates

**Figure 3:**
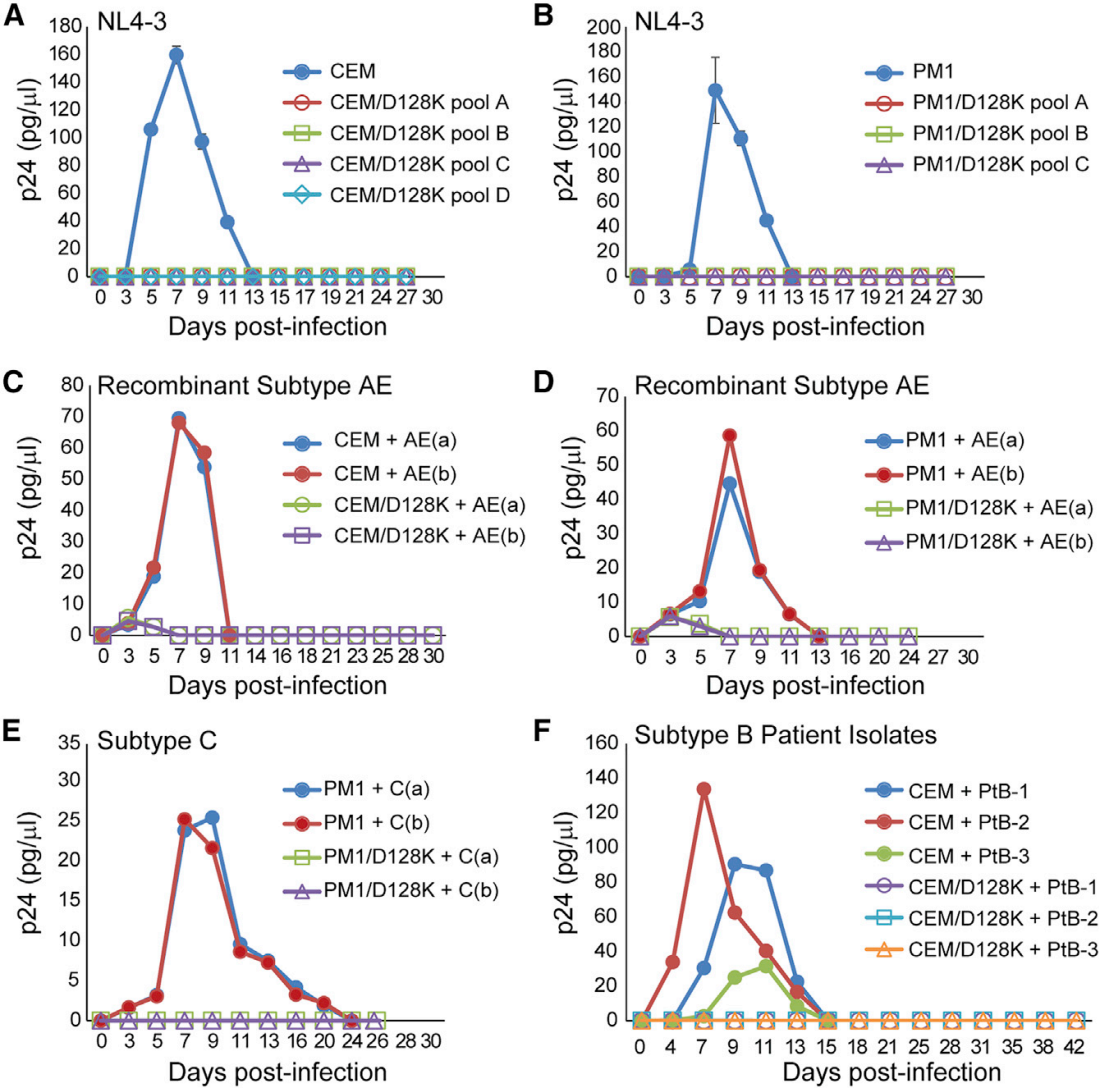
Replication Kinetics of Group M Viruses in CEM/D128K and PM1/D128K Cell Lines Normalized virus from subtype B (NL4-3), recombinant subtype AE, and subtype C (MJ4) virus and patient-derived subtype B isolates were used to infect parental and A3G-D128K-expressing T cell lines. Target cells were infected with virus (~70 pg p24 CA/1 × 10^6^ cells), supernatants were harvested every 2 to 3 days, and p24 CA amounts were determined by ELISA. Multiple rounds of infection were monitored up to 42 days post-infection. (A and B) Replication kinetics of NL4-3 in parental (A) CEM and (B) PM1 cells, with no detectable virus 27 days post-infection in 4 and three independent CEM/D128K and PM1/D128K cell lines, respectively. The averages of replication kinetics from duplicate flasks are indicated. Error bars represent SD. (C and D) Replication kinetics of recombinant subtype AE in parental (C) CEM and (D) PM1 cells, with no detectable virus up to 30 days post-infection in CEM/D128K and PM1/D128K cell lines. Replication kinetics from duplicate flasks are indicated for each cell line. (E) Replication kinetics of CCR5-tropic subtype C clone MJ4 in parental PM1 cells, with no detectable virus in PM1/D128K cells 26 days post-infection. Replication kinetics from duplicate flasks are indicated. (F) Replication kinetics of three subtype B patient isolates (PtB-1, -2, and -3) in CEM cells. No detectable virus was measured in the CEM/D128K cell line 42 days post-infection. The averages of replication kinetics from duplicate flasks are indicated.

To determine whether stable expression of A3G-D128K inhibited HIV-1 replication and spread, 4 CEM/D128K pools and three PM1/D128K pools (generated from independent transductions with A3x3G(DK) virus) were infected with equivalent p24 CA amounts of NL4-3 virus, and virus replication and spread were monitored by determining the amount of p24 CA in the culture supernatant every 2 to 3 days for 27 days (Figures 3A and 3B). NL4-3 virus replicated in the parental CEM and PM1 cells, and p24 CA amounts peaked at 7 days post-infection. However, no NL4-3 replication was detected in the CEM/D128K and PM1/D128K cells for 27 days, indicating that A3G-D128K expression potently inhibited NL4-3 replication. Furthermore, no A3G-D128K-resistant viral variants emerged in 7 independent cultures for 27 days. Similar inhibition of replication of an CXCR4-tropic subtype AE recombinant (90CF402.1.8) was observed in the CEM/D128K and PM1/D128K cell pools for 24–30 days (Figures 3C and 3D, respectively). Inhibition of replication of CCR5-tropic subtype C virus from molecular clone MJ4 in the PM1/D128K cells was also observed for 26 days (Figure 3E), indicating that A3G-D128K expression in T cells can potently inhibit the replication and spread of multiple subtypes, including highly prevalent subtypes B, C, and AE recombinant.

We sought to determine whether A3G-D128K-resistant variants can be selected in culture from a genetically diverse population of viruses derived from patients. Three patient-derived subtype-B isolates that were previously characterized to be CXCR4-tropic^49^ were obtained, and their ability to replicate in parental CEM cells and CEM/D128K cells was determined (Figure 3F). Although all three patient isolates replicated in the parental CEM cells with kinetics similar to that of the NL4-3 virus, with p24 CA levels peaking around day 7 (PtB-2) or days 9–11 (PtB-1 and PtB-3), no viral replication was detected in CEM/D128K cells for 42 days (Figure 3F). The results indicated that A3G-D128K expression was able to restrict the replication of genetically diverse populations of HIV-1 isolated from subtype-B-infected patients.

### CEM/D128K and PM1/D128K Cells Can Support Replication of HIV-2 and SIVmac239

To confirm that the lack of HIV-1 replication and spread in CEM/D128K and PM1/D128K cell pools was not due to the inadvertent selection of cell variants that cannot support viral replication, CEM/D128K and PM1/D128K cell lines were infected with normalized inputs of wild-type HIV-2 or SIVmac239 virus, which express Vif proteins that can degrade A3G-D128K.^45^ SIVmac239 and HIV-2 viruses replicated in the CEM/D128K and PM1/D128K cell lines (Figure S4), indicating that both cell lines can support the replication and spread of SIVmac239 and HIV-2 but not HIV-1.

### Inhibition of HIV-1 Replication Is Dependent on the Proportion of Target Cells that Express A3G-D128K

**Figure 4:**
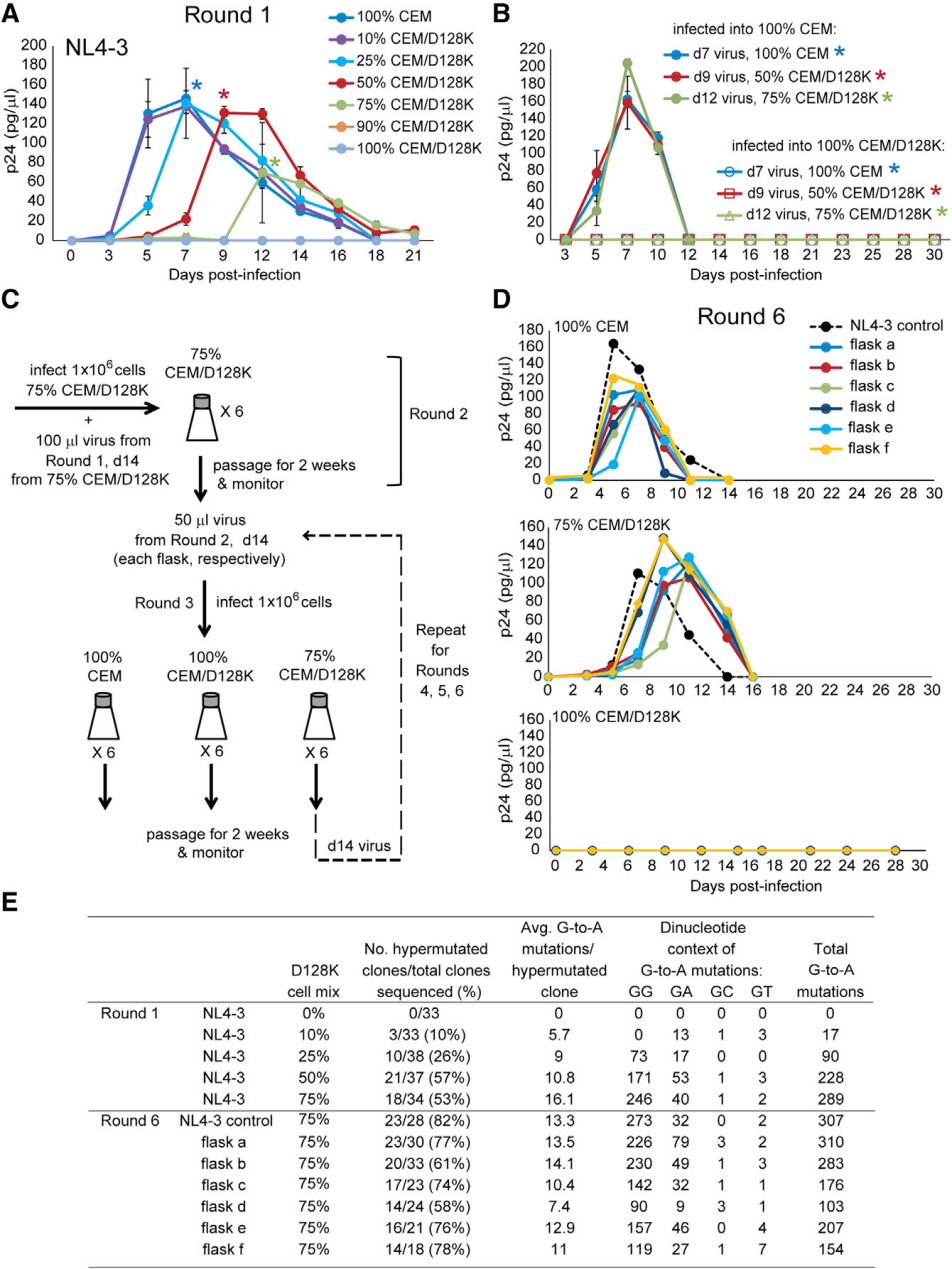
Absence of Selection for NL4-3 Variants that Can Replicate in CEM/D128K Cells (A) NL4-3 virus was used to infect different ratios of CEM:CEM/D128K cells. 100%, 90%, 75%, 50%, 25%, 10%, and 0% CEM cells were mixed with 0%, 10%, 25%, 50%, 75%, and 100% CEM/D128K cells, respectively. Supernatants were collected every 2 to 3 days for 21 days and measured by p24 CA ELISA to determine replication kinetics. Average of two independent infections per ratio is indicated. Error bars represent SD. (B) Viruses from peak production, normalized for p24 CA, from round 1 cultures 100% CEM (day 7; indicated by a blue star in A), 50%CEM:50%CEM/D128K (day 9; indicated by a red star in A), and 25% CEM:75% CEM/D128K (day 12; indicated by a green star in A) were used to infect either 100% CEM or 100% CEM/D128K cells. Supernatants were collected every 2 to 3 days for up to 30 days, and p24 CA amounts were quantified by ELISA to determine replication kinetics. Infection of the parental CEM cells showed wild-type NL4-3 peak virus at day 7 with no detectable virus measured in 100% CEM/D128K cells. Averages of two independent infections per cell ratio are indicated. Error bars represent SD. (C) Protocol to select for HIV-1 resistance to A3G-D128K. (D) Round 6 replication kinetics. Viruses from round 6 (independent flasks a–f) were used to infect 100% CEM, 25% CEM:75% CEM/D128K, or 100% CEM/D128K cells, and viral supernatants were collected every 2 to 3 days for up to 28 days. ELISA was used to quantify p24 CA to determine replication kinetics. Viruses from flasks a–f displayed wild-type kinetics in parental CEM cells peaking at days 5–7 and showed delayed kinetics, peaking at days 9–12, in the 25% CEM:75% CEM/D128K cell mix as was seen in round 1, as indicated in (A). No detectable virus was measured in 100% CEM/D128K cells. (E) Proviral DNA from cell cultures in which virus replication peaked in round 1 and round 6 were analyzed for G-to-A hypermutation by sequencing an 896-nt Vif region. For round 1, proviral DNAs from peak virus day 7 for 0%, 10%, and 25% CEM/D128K; day 9 for 50% CEM/D128K; and day 12 for 75% CEM/D128K were analyzed. Number of clones is combined from 2 independent infections; an 896-nt Vif region was sequenced. For round 6, proviral DNA from cell cultures in which virus replication peaked (day 9) was analyzed for 75% CEM/D128K from 6 independent flasks a–f. Error bars represent SD.

To determine how the proportion of target cells that express A3G-D128K affect inhibition of virus replication, we generated mixtures of CEM and CEM/D128K cells at different ratios, ranging from 0% to 100% CEM/D128K cells, and monitored the kinetics of the replication of NL4-3 in the cell mixtures for 21 days (Figure 4A). NL4-3 replication peaked on day 7 in cultures that had 0% or 10% CEM/D128K cells and peaked on days 7–9, 9–12, and 12–14 in cultures that had 25%, 50%, and 75% CEM/D128K cells, respectively. Virus replication was not detected in cultures that had 90% or 100% CEM/D128K cells. Thus, A3G-D128K expression in a subpopulation of target cells delayed viral replication kinetics in a dose-dependent manner.

Next, viruses harvested on days of peak viremia from cultures containing 0%, 50%, and 75% CEM/D128K cells were used to infect either 100% CEM cells or 100% CEM/D128K cells (Figure 4B). In all cases, the viruses replicated with wild-type kinetics in CEM cells, indicating the presence of infectious virions, but no virus replication was detected in CEM/D128K cells, indicating that A3G-D128K-resistant viral variants did not emerge. PCR amplification and sequencing of genomic DNA from cells on days of peak virus production revealed extensive G-to-A hypermutations from cell cultures in which >25% of the cells expressed A3G-D128K (Figure 4E, round 1). Furthermore, the proportion of hypermutated viral DNAs increased from 0/33 to 18/34 in cultures in which 75% of the cells expressed A3G-D128K (p < 0.005, Fisher’s exact test). Thus, inhibition of virus replication was correlated with A3G-D128K expression and its antiviral activity in a subpopulation of target cells.

### No Detectable A3G-D128K-Resistant Virus after Multiple Rounds of Replication in CEM/D128K Cells

In an effort to select for A3G-D128K-resistant viral variants, we passaged the NL4-3 virus for ∼3 months in 25% CEM:75% CEM/D128K mixed-cell cultures, which supported viral replication with a 5-day delay in peak virus production (Figure 4A). Six rounds of infections were carried out by passaging the virus for 2 weeks during each round and using the culture supernatant at the peak of virus production to initiate the next round of infection (Figure 4C). Virus from the peak of virus production in the initial 25% CEM:75% CEM/D128K culture (Figure 4A, round 1) was used to infect 6 flasks (labeled a–f) containing fresh 25% CEM:75% CEM/D128K mix and cultured for 2 weeks (Figure 4C, round 2). This protocol was repeated for rounds 3–6, and the results of infection of 100% CEM, 75% CEM/D128K, and 100% CEM/D128K cells with virus from round 6 are shown in Figure 4D. Virus replication was not detected in 100% CEM/D128K cultures after infection with virus from rounds 1–6, indicating that A3G-D128K-resistant virus did not emerge after >3 months of culture. In all cases, virus replication with wild-type NL4-3 kinetics was detected in 100% CEM cells, and delayed replication kinetics were observed in the 25% CEM:75% CEM/D128K cell mix, confirming the presence of replicating virus in the cultures. Analysis of integrated proviruses at the time point of peak virus production from the 25% CEM:75% CEM/D128K cell mix infected with round 6 virus showed that >50% (up to 78%) of the integrated proviruses were hypermutated, with an average of 7–14 G-to-A changes per hypermutated clone in the 896-nt Vif region (Figure 4E, round 6). Overall, these results indicated that CD4^+^ T cell lines expressing A3G-D128K were capable of long-term HIV-1 inhibition without detectable emergence of A3G-D128K-resistant virus.

### Expression of A3G-D128K in CEMSS Cell Pools and Clones Potently Inhibits HIV-1 Replication without Detectable Emergence of Resistant Virus

**Figure 5:**
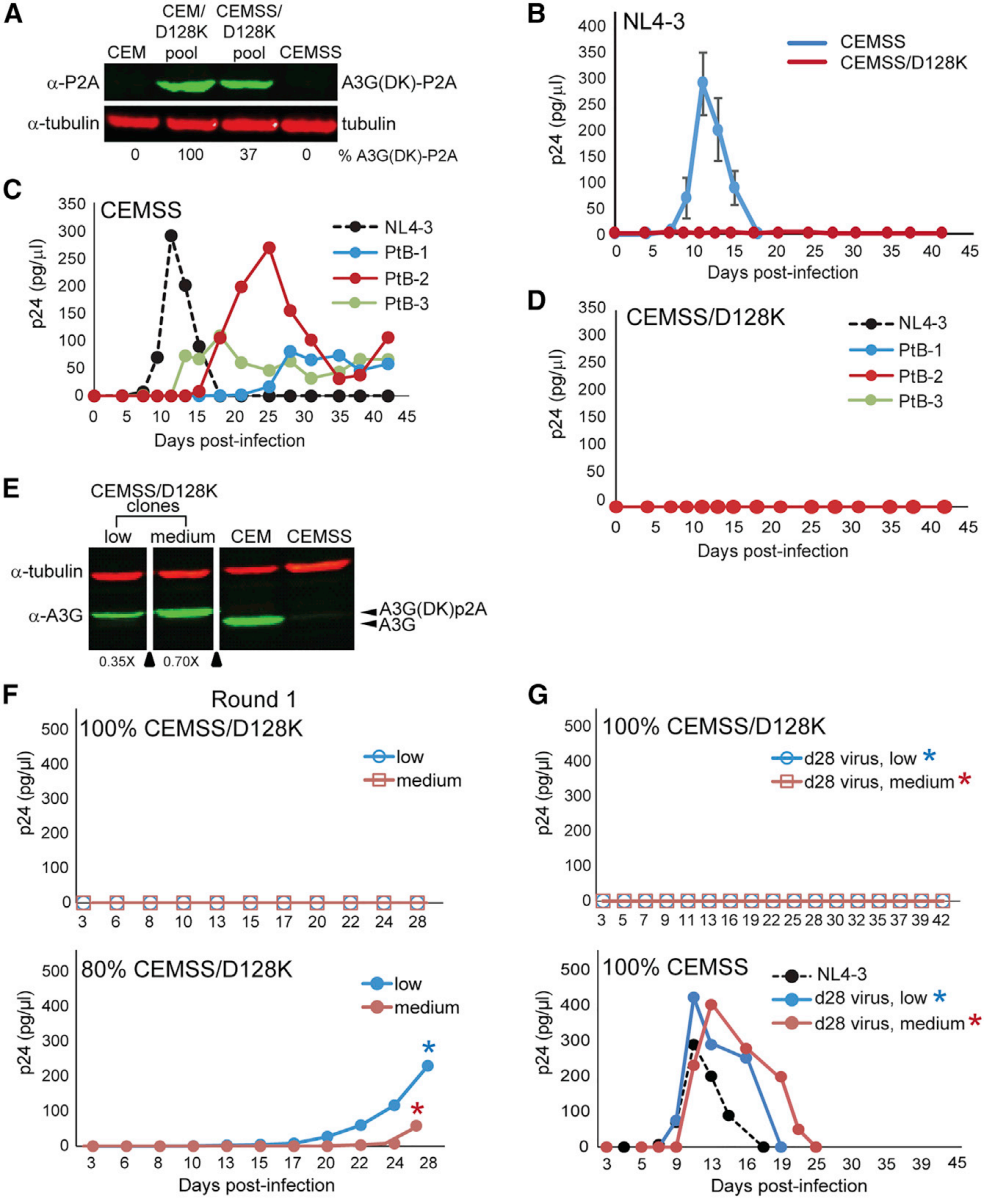
Absence of Selection for NL4-3 Variants that Can Replicate in CEMSS/D28K Cells (A) CEMSS cells were transduced with A3x3G(DK) virus and selected for puromycin resistance. Western blot analysis shows the level of A3G-D128K-P2A expressed in the CEMSS/D128K cell pool (37%) compared to the level in the CEM/D128K cell pool (set to 100%) and normalized to α-tubulin. Blot probed with α-P2A (green) and α-tubulin (red). (B) CEMSS/D128K and parental CEMSS cells were infected with NL4-3 (70 pg p24 CA/1 × 10^6^ cells). Supernatants were harvested every 2 to 3 days, and p24 CA amounts were determined by ELISA. Multiple rounds of infection were monitored over a 40-day period. NL4-3 shows wild-type kinetics with peak virus at day 11, with no detectable virus in CEMSS/D128K cells. Averages of two independent infections are indicated. Error bars represent SD. (C and D) Equal amounts of p24-containing virus from NL4-3 and three subtype B patient isolates (PtB-1, -2, and -3) were used to infect (C) CEMSS and (D) CEMSS/D128K cell lines, respectively. Supernatants were harvested every 2 to 3 days for 42 days and monitored by ELISA for p24 CA expression. Averages of replication kinetics of two independent infections are shown. Error bars represent SD. (E) Western analysis of puromycin-resistant CEMSS/D128K single-cell clones expressing A3G-D128K. Two CEMSS/D128K clones are shown with lower A3G-D128K levels (0.35× and 0.70×) compared to endogenous A3G expressed in CEM cells (1×). Blot probed with α-A3G (green) and α-tubulin (red). (F) Replication kinetics of NL4-3 in CEMSS/D128K clones (100%) or a mix of 20% CEMSS:80% CEMSS/D128K low (blue) or medium (red) clones. Viral supernatants were collected every 2 to 3 days, and p24 CA was measured by ELISA up to 28 days post-infection. No NL4-3 virus was detected in the 100% CEMSS/D128K cells (top). Severely delayed kinetics were observed in the 20% CEMSS:80% CEMSS/D128K clone mix 28 days post-infection (bottom). (G) Normalized virus from day 28, round 1, 20% CEMSS:80% CEMSS/D128K cells (low and medium, indicated by blue and red stars, as also shown in the lower panel of F) was used to infect 100% CEMSS/D128K or 100% CEMSS cells. Viral supernatants were collected up to 42 days post-infection and analyzed for p24 CA by ELISA. No NL4-3 replication was detected in 100% CEMSS/D128K cell lines, and near-wild-type kinetics (days 11–13) were observed in 100% CEMSS cells.

CEM/D128K cells express endogenous A3G and A3F, but CEMSS cells do not. We hypothesized that A3G-D128K-resistant virus might emerge more efficiently in the absence of expression of endogenous A3G and A3F, since Vif would not need to retain the ability to induce degradation of the endogenous A3 proteins. To test this hypothesis, we generated CEMSS cells that stably expressed A3G-D128K by transduction with the A3x3G(DK) lentivector and selecting a pool of puromycin-resistant cells (CEMSS/D128K). Western blotting analysis showed that the CEMSS/D128K cell pool expressed A3G-D128K at 37% of the level observed with CEM/D128K (Figure 5A) and that expression of A3G-D128K did not alter the growth kinetics of the CEMSS cells (Figure S3C). We infected the parental CEMSS and CEMSS/D128K cells with equivalent p24 CA amounts of NL4-3 virus and monitored HIV-1 replication kinetics (Figure 5B). The results showed that NL4-3 replication was readily detected in the CEMSS cells, but no virus replication was detected in the CEMSS/D128K cells for 42 days, indicating that resistance to A3G-D128K did not emerge after prolonged culture, even in the absence of endogenous A3 proteins. Similar to the results observed for CEM/D128K cells (Figure 3F), infection of CEMSS/D128K cells with subtype-B-derived patient isolates did not result in the detectable emergence of A3G-D128K-resistant virus (Figures 5C and 5D).

To determine whether resistant virus would emerge in CEMSS/D128K cells expressing lower amounts of A3G-D128K, single-cell clones were selected that expressed A3G-D128K-P2A at a 35% (low) or a 70% (medium) level compared to endogenous A3G expressed in CEM cells (Figure 5E). Both CEMSS/D128K cell clones were infected with NL4-3 and passaged for 28 days. As seen with CEM/D128K cells, the CEMSS/D128K cell clones also inhibited productive viral replication (Figure 5F, top). However, NL4-3 infection of a 20% CEMSS:80% CEMSS/D128K cell mix for each cell clone showed severely delayed kinetics, and virus production did not become detectable for 20 days after infection (Figure 5F, bottom). Viruses from the 20% CEMSS:80% CEMSS/D128K (low or medium) cultures were used to infect 100% CEMSS/D128K cell clones and monitored for viral replication and spread (Figure 5G, top). No virus replication was detected after 42 days, indicating that A3G-D128K-resistant virus did not emerge, even though replication-competent virus was detected upon infection of the parental CEMSS cells (Figure 5G, bottom). Overall, expression of A3G-D128K in the absence of endogenous A3 proteins did not lead to the emergence of A3G-D128K-resistant viral variants.

### HIV-1 Vif ^14^SEMQ^17^ Mutant Replicates with Severely Delayed Kinetics in A3G-D128K-Expressing Cells

**Figure 6:**
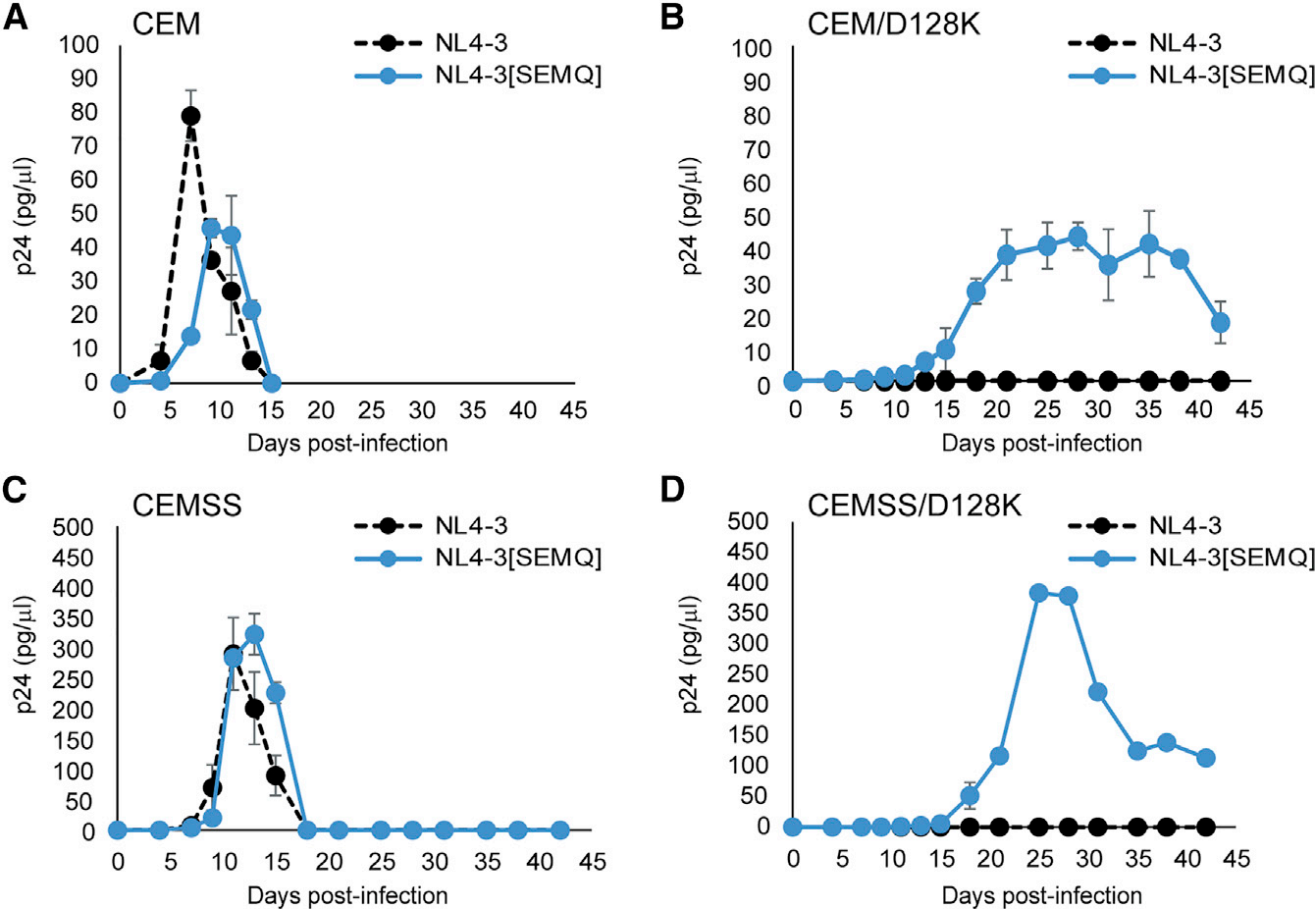
Replication Kinetics of the NL4-3 Vif ^14^SEMQ^17^ Mutant in CEM/D128K and CEMSS/D128K Cell Lines (A–D) Normalized virus from NL4-3 and NL4-3[SEMQ] was used to infect (A) CEM, (B) CEM/D128K, (C) CEMSS, and (D) CEMSS/D128K cell lines. Supernatants were harvested every 2 to 3 days for up to 42 days and monitored by ELISA for p24 CA expression. Averages of replication kinetics from two independent infections are indicated. Error bars represent SD.

It was previously reported that substitution of HIV-1 Vif amino acids ^14^DRMR^17^ with African green monkey amino acids ^14^SE(R/M)Q^17^ conferred the ability to induce degradation of A3G-D128K.^50^ However, the HIV-1 Vif ^14^SEMQ^17^ variant is unable to induce degradation of human A3F in single replication cycle assays.^51^^,^^52^ To determine whether the HIV-1 Vif ^14^SEMQ^17^ mutant can replicate in A3G-D128K expressing cells, we constructed an NL4-3 mutant in which the Vif amino acids ^14^DRMR^17^ were replaced with ^14^SEMQ^17^ (NL4-3[SEMQ]), which also resulted in the substitution of integrase (IN) C-terminal 5 amino acids from RQDAD to KRDAD. We generated NL4-3[SEMQ] virus and infected the parental CEM and CEMSS as well as CEM/D128K and CEMSS/D128K cells (Figure 6). In the parental CEM and CEMSS cells, the NL4-3[SEMQ] peak virus production was delayed by by 2–3 days compared to wild-type NL4-3, suggesting that the SEMQ mutant may have a lower fitness than wild-type NL4-3 (Figures 6A and 6C). In CEM/D128K or CEMSS/D128K cells, wild-type NL4-3 replication was not detected for 42 days, whereas NL4-3[SEMQ] replicated with severely delayed kinetics (Figures 6B and 6D). These results suggest that the NL4-3[SEMQ] mutant can overcome the antiviral activity of A3G-D128K, but it either is less efficient at inducing A3G-D128K degradation or has a lower fitness than wild-type NL4-3.

### Efficient Transduction of CD4^+^ T Cells and CD34^+^ HSPCs with A3x3G-D128K-Expressing Lentivector

**Figure 7:**
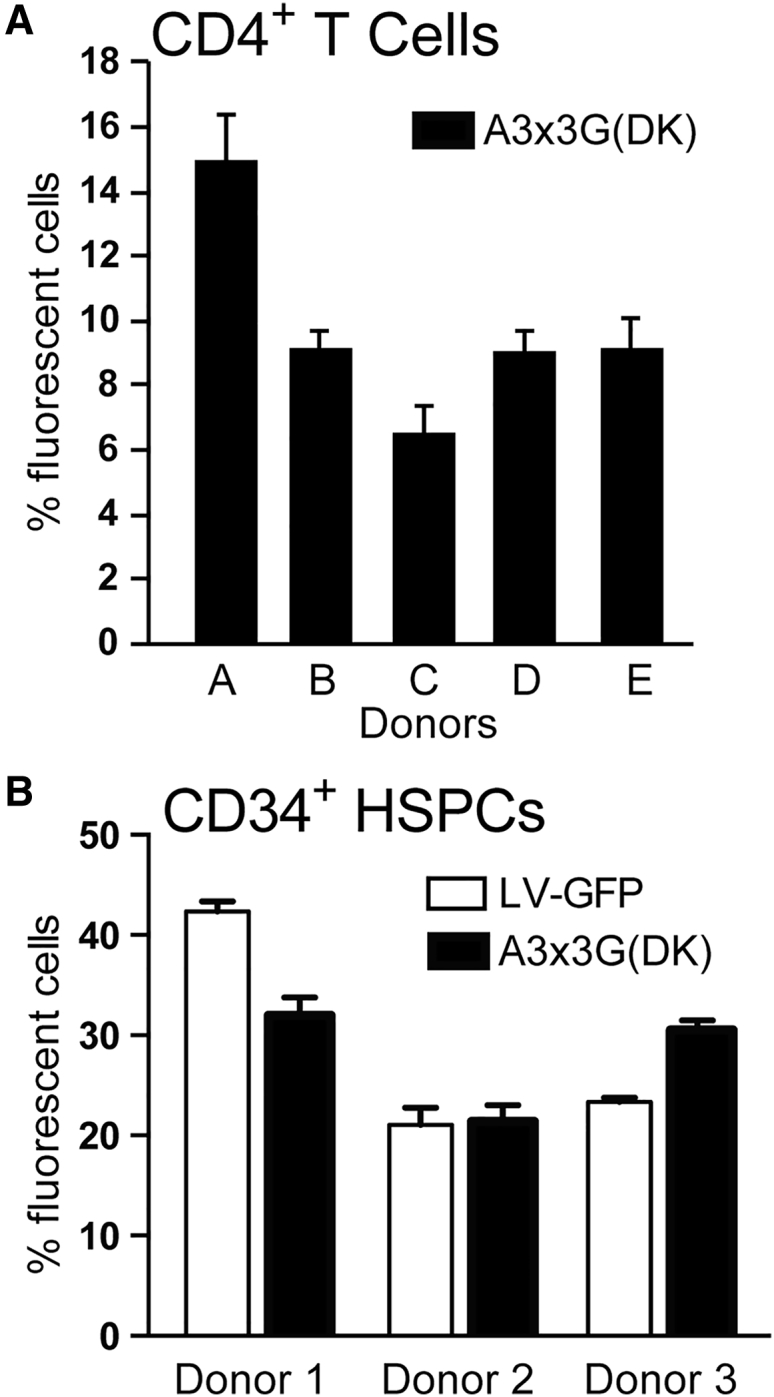
Efficient Transduction of CD4^+^ T Cells and CD34^+^ HSPCs with the A3x3G-D128K-Expressing Vector (A) CD4^+^ T cells from 5 donors were transduced with A3x3G(DK) vector virus at an MOI of 10, and the proportion of eYFP^+^ cells was determined by FACS 72 h post-infection. (B) CD34^+^ HSPCs from 3 donors were transduced with A3x3G(DK) vector virus or a control GFP lentiviral vector (LV-GFP) at an MOI of 20, and the proportion of fluorescent cells was determined by FACS 6 days post-transduction. Average from two independent infections is indicated. Error bars represent SD.

It is desirable to transduce activated CD4^+^ T cells, the natural targets of HIV-1 infection, and CD34^+^ HSPCs, which can differentiate to form new CD4^+^ T cells. CD4^+^ T cells from 5 different donors (Figure 7A) were transduced with the A3x3G(DK) lentivector (MOI = 10), resulting in the expression of eYFP in 9%–15% of the CD4^+^ T cells. Transduction of CD34^+^ HSPCs from three different donors with the A3x3G(DK) lentivector (MOI = 20) resulted in eYFP expression in 32% of HSPCs, and transduction with the LV-GFP lentivector resulted in GFP expression in ∼40% of the HSPCs (Figure 7B); no apparent cytotoxicity was observed (data not shown). These results confirmed that the A3x3G(DK) lentivector can efficiently transduce activated CD4^+^ T cells and CD34^+^ HSPCs.

## Discussion

Two patients have remained free of HIV-1 in the absence of therapy for >18 months to several years after HSPC transplantation of donor cells with a Δ32 deletion in the CCR5 coreceptor, suggesting that inactivating the CCR5 coreceptor using gene therapy might achieve a cure.^3^^,^^4^^,^^53^ However, inactivation of the *CCR5* does not prevent infection with CXCR4-tropic and dual-tropic viruses and is likely to select for variants that can utilize the CXCR4 coreceptor,^8^^,^^54^ suggesting a need for strategies to suppress infection with CCR5-, CXCR4-, and dual-tropic viruses. Other gene therapy strategies that are being explored to achieve a functional cure that can potentially inhibit replication of all viruses, regardless of the coreceptor they utilize, include expression of a fusion peptide,^55^ genetically modified chimeric antigen receptor (CAR)-expressing T cells,^56^ deletion of HIV-1 proviruses by gene editing,^57^^,^^58^ and harnessing the antiviral activity of host restriction factors.^59^, ^60^, ^61^

In the studies reported here, we developed self-activating lentiviral vectors that suppress A3G-D128K expression in the virus-producing cells and efficiently reconstitute a functional *A3G-D128K* by homologous recombination during reverse transcription. Consequently, >70% of the target 293T cells were transduced with the A3x3G(DK) vector, compared to only 8% transduction efficiency with the control A3G(DK) vector. Importantly, none of the 52 proviral DNAs from cells transduced with the A3x3G(DK) virus produced in the presence of Vif2 were hypermutated, indicating that the A3x3G(DK) vectors were very effective in suppressing A3G-D128K expression in the virus-producing cells. Our observed high efficiency of homologous recombination (89%–98%) for the ∼900-bp direct repeat sequence of A3G is consistent with previous observations by us and others that a 701-bp direct repeat and a 971-bp direct repeat were deleted at frequencies of 91% and 81%, respectively.^35^^,^^38^^,^^39^ Intriguingly, we were unable to select for A3G-D128K-resistant viral variants in multiple viral cultures for > 3.5 months, suggesting that A3G-D128K has a high genetic barrier to development of resistance. One possible reason for the high genetic barrier is that the G-to-A hypermutation induced by A3G is a lethal “all-or-nothing” phenomenon.^62^ Hypermutated proviruses in infected patients contain, on average, 231 G-to-A substitutions, of which ∼47 result in stop codons; thus, the probability of a virus escaping A3G-mediated inactivation by sublethal mutagenesis is <10^−21^.^62^ We postulate that multiple amino acid substitutions in Vif must appear simultaneously to acquire the ability to induce A3G-D128K degradation. If the appearance of one or two of the amino acid changes does not confer complete resistance to A3G-D128K, the all-or-nothing nature of hypermutation predicts that these intermediate substitution mutants would be quickly eliminated from the viral population. The probability of acquiring three specific nucleotide substitutions in a single viral genome during one replication cycle is the product of the rate for each substitution, which is 3 × 10^−5^/nucleotide/replication cycle: thus, the probability of acquiring three specific substitutions is (3 × 10^−5^) × (3 × 10^−5^) × (3 × 10^−5^) or 2.7 × 10^−14^.^43^^,^^63^^,^^64^ Thus, because of the relatively small size of the viral populations in the cell culture assays and the limited number of replication cycles (∼100, assuming one replication cycle per day), it is unlikely that a mutant with three simultaneous amino acid changes will appear in our selection experiments.

A3G-D128K-resistant variants did not emerge in CEMSS/D128K cells, which did not express other A3 proteins, suggesting that the high genetic barrier to resistance is intrinsic to the all-or-nothing nature of lethal hypermutation. Consistent with our results, a computational study predicted that the most potent therapeutic anti-HIV-1 A3G-based strategy is to use a Vif-binding site mutant.^65^ Previous efforts to select for A3G-resistant Vif-defective viral variants have yielded negative results or mutations that were unable to confer the ability to replicate in A3G-expressing T cell lines.^66^, ^67^, ^68^ In a recent study, Vif null viral variants with Env substitutions were selected, but these mutations did not confer A3F resistance, suggesting that the mechanism of resistance may be specific to the level of A3G expression.^67^

Potential benefits of gene therapy with Vif-resistant A3G-D128K are that CCR5-tropic as well as CXCR4-tropic viruses will be inhibited, and that it is possible to express more than one Vif-resistant A3 protein to inhibit viral replication and spread. Distinct Vif-A3G and Vif-A3F interactions are necessary for inducing degradation of the A3 proteins,^51^^,^^69^^,^^70^ indicating that multiple Vif mutations may be required to overcome different Vif-resistant A3 proteins. A3G-D128K differs from wild-type human A3G by a single amino acid, which should minimize elimination of the genetically engineered cells by the host immune system. *In vivo* patient studies of elite controllers have been shown to not only attenuate Vif proteins^71^ but also increase A3G mRNA and protein levels, leading to lower HIV provirus burden, emphasizing the importance of Vif-APOBEC3 interactions in controlling viral loads.^72^^,^^73^

Although our results indicate that efficient lentivector delivery of Vif-resistant A3G to HIV-1 target cells is a promising anti-HIV gene therapy, additional technical developments and testing in a humanized mouse model system will be needed before its implementation in the clinic. First, it will be necessary to demonstrate that a sufficiently high proportion of primary CD4+ T cells, the target of HIV-1 infection, can be engineered to express A3G-D128K to effectively inhibit HIV-1 replication and spread. Our cell-mixing experiments in which parental CEM and CEM/D128K cells were mixed at different ratios suggest that A3G-D128K expression in >75% of the target cells may be needed to significantly inhibit viral replication and spread. Our efficiency of transduction of the activated CD4+ T cells was lower than the efficiencies reported in recent studies but could be improved by adapting recently improved transduction methods.^74^ In future studies, it may be possible to achieve high levels of lentiviral transduction as well as enable pharmacologic selection of the transduced cells. Second, the A3G-D128K-expressing cells will be susceptible to infection by HIV-1, resulting in their elimination from the patients. It may be possible to provide the A3G-D128K-expressing cells with a selection advantage over non-expressing cells by co-expression of anti-CCR5 shRNAs or a fusion peptide that inhibits HIV-1 entry.^55^ Successful combination of A3G-D128K expression with elements that provide a selection advantage to the transduced cells, as well as achieving a high efficiency of lentivector transduction, may establish the feasibility of HIV-1 gene therapy with Vif-resistant A3G.

In summary, we constructed and characterized self-activating lentiviral vectors that can efficiently deliver Vif-resistant A3G-D128K to a high proportion of CD4^+^ T cells and CD34^+^ HSPCs. Additional improvements in lentivector transduction efficiency, providing the A3G-D128K-expressing cells with a selective advantage, and testing the efficacy of this anti-HIV gene therapy in an animal model system will help determine whether gene therapy with Vif-resistant A3G can provide a viable anti-HIV treatment and functional cure strategy.

## Materials and Methods

### Plasmid Construction

Plasmids used in this study are designated with a “p,” while the names of viruses and proviruses generated from these plasmids are not. pC-Help is an HIV-1 helper construct that lacks a packaging signal and primer-binding site; the helper construct expresses all the viral proteins except Nef and envelope,^75^ and pHCMV-G expresses the G glycoprotein of vesicular stomatitis virus (VSV-G).^76^ pVif2-HA expresses codon-optimized hemagglutinin (HA)-tagged Vif from HIV-2.^77^ pcDNA-APO3G_D128K_ expresses A3G mutant D128K^45^ and was used to PCR-amplify the A3G-D128K sequences used for construction of other vectors. pHL[WT] is an HIV-1 luciferase-encoding vector that expresses all the HIV-1 proteins except Nef and envelope.^78^ pNL4-3 expresses a replication-competent subtype B HIV-1.^79^ pMJ4 is a subtype C HIV-1 infectious molecular clone, obtained through the NIH AIDS Reagent Program, Division of AIDS, National Institute of Allergy and Infectious Diseases (NIAID), NIH (from Drs. Thumbi Ndung’u, Boris Renjifo, and Max Essex; catalog #6439).^80^ p90CF402.1 is an intersubtype recombinant AE HIV-1 infectious molecular clone, obtained through the NIH AIDS Reagent Program, Division of AIDS, NIAID, NIH (from Drs. Feng Gao and Beatrice Hahn; catalog #3284).^81^ pROD12 is a full-length infectious molecular clone of HIV-2 (a gift from Keith Peden). pSIVmac239 is an infectious clone of simian immunodeficiency virus (SIV), obtained through the NIH AIDS Reagent Program, Division of AIDS, NIAID, NIH (from Dr. Ronald C. Desrosiers; catalog #12249).^82^ Subtype B patient isolates were obtained through the NIH AIDS Reagent Program, Division of AIDS, NIAID, NIH from the panel of 60 international HIV-1 isolates (catalog #11412),^49^ contributor Dr. Robert Gallo for HIV-1 MN virus (catalog #317),^83^ and contributor Dr. Nelson Michael for HIV-1 BK132 (GS 009) virus (catalog #7691) and HIV-1 BZ167/GS 010 (89BZ_167) virus (catalog #7692).^84^ For clarity, HIV-1 MN virus was labeled as PtB-3, HIV-1 BK132 (GS 009) virus was labeled as PtB-1, and HIV-1 BZ167/GS 010 (89BZ_167) virus was labeled as PtB-2. pNL4-3[SEMQ] was constructed by replacing the Vif ^14^DRMR^17^ with ^14^SEMQ^17^ in NL4-3. Since the first 19 amino acids of Vif overlap with the C-terminal region of IN, substitution of ^14^DRMR^17^ with ^14^SEMQ^17^ changed the 5 terminal amino acids of IN from RQDAD to KRDAD. These substitutions were previously shown to have a minimal effect on virus replication.^85^ Plasmid pLV-GFP is a ubiquitin-C-driven EGFP-expressing vector (catalog #14884, Addgene, Watertown, MA, USA).

The multiple cloning site 5′ of the IRES-puromycin cassette in the lentiviral vector pLVX-IRES-Puro (catalog #632183; Clontech, Mountain View, CA, USA) was utilized to construct the following vectors. Vector peYFPip expresses *eYFP* and *puro*. Vector pA3G(DK) was created by insertion of A3G-D128K lacking a stop codon followed by an in-frame insertion of P2A fused to eYFP lacking a start codon. Insertion of the 5′ direct repeat fragment of A3G-D128K (nt 1 to 1,083) followed by an in-frame insertion of P2A fused to eYFP lacking a start codon created vector pA3dr(DK). Insertion of the 3′ direct repeat fragment of A3G-D128K (nt 183 to 1,152) followed by an in-frame insertion of P2A fused to eYFP lacking a start codon created vector p3Gdr(DK). To create vectors pA33G(DK) and pA3x3G(DK), the following fragments were joined by ligation in the same reading frame: the 5′ direct repeat of A3G-D128K (1 to 1,083 nt of A3G for the A33G(DK) vector and the same 1 to 1,083 nt plus three in-frame stop codons, TGA-TAA-TGA for the A3x3G(DK) vector); 3′ direct repeat of A3G-D128K (nt 184 to 1,152); and P2A fused to eYFP (lacking a start codon). This vector has an ∼900-bp direct repeat of A3G-D128K (nt 184 to 1,083).

To create pCMV-A3G(DK), pCR3.1(+) (catalog #V79020; Invitrogen, Carlsbad, CA, USA) was digested with Nhe I and Xba I, and A3G-D128K was amplified from pA3G(DK) with Nhe I-containing forward primer and Xba I-containing reverse primers for complementary sticky-end ligation. To create pCMV-A3G(DK)-P2A-eYFP, the Nhe I and Xba I cloning sites were used to insert A3G-D128K lacking a stop codon, and the Xba I and Apa I sites were used to ligate an in-frame fragment of P2A fused to eYFP (lacking a start codon). The structures of all final plasmids were confirmed by sequencing (Macrogen, Rockville, MD, USA).

### Tissue Culture and Cell Lines

Cell lines 174XCEM (NIH AIDS Reagent Program, Division of AIDS, NIAID: 174XCEM cells from Dr. Peter Cresswell; CD4^+^/CXCR4^+^/CCR5^−^/GPR15^+^ [catalog #272]),^86^ H9 (T-lymphoid cell line, American Type Culture Collection, catalog #HTB-176), PM1 (NIH AIDS Reagent Program, Division of AIDS, NIAID: PM1 from Dr. Marvin Reitz; derived from HUT 78 cells; catalog #3038),^87^ and CEMSS (NIH AIDS Reagent Program, Division of AIDS, NIAID: CEM-SS from Dr. Peter L. Nara; catalog #776)^88^ were grown in RPMI 1640 medium (CellGro, Manassas, VA, USA) supplemented with 10% fetal calf serum (HyClone, Logan, UT, USA) and 1% penicillin-streptomycin (final concentration: penicillin, 50 U/mL; and streptomycin, 50 μg/mL; Lonza, Walkersville, MD, USA). HEK293T cells (American Type Culture Collection; catalog #CRL-3216) and TZM-bl cells (NIH AIDS Reagent Program, Division of AIDS, NIAID, NIH: TZM-bl from Dr. John C. Kappes, Dr. Xiaoyun Wu, and Tranzyme [catalog #8129])^89^^,^^90^ were grown in DMEM (CellGro) supplemented with 10% fetal calf serum (HyClone) and 1% penicillin-streptomycin stock (final concentration: penicillin, 50 U/mL; and streptomycin, 50 μg/mL; Lonza). All cells were maintained in humidified 37°C incubators with 5% CO_2_.

Umbilical cord blood (UCB) CD34^+^ cells were isolated and verified as described previously from cord blood generously donated from the Cleveland Cord Blood Center (Cleveland, OH, USA).^91^ All approved human protocols are available upon request at The Scripps Research Institute.

### Transfection, Virus Production, and Single-Cycle Transduction Assays

All transfections were performed using TransIT-LT1 Transfection Reagent (Mirus Bio, Madison, WI, USA) according to the manufacturer’s instructions. To generate virus, 293T cells were seeded at 3 × 10^6^ cells per 100-mm dish and transfected with pC-Help (5 μg), pHCMV-G (4 μg), and vectors of interest (10 μg). Twenty-four to 30 h post-transfection, virus was harvested, filtered with 0.45-μm filters, and stored at −80°C. For generating A33G(DK) or A3x3G(DK) virus, pVif2-HA (2–4 μg) was also included in the transfection to degrade any expressed A3G-D128K in the producer cells as a result of direct repeat deletion during transfection. Direct repeat deletion during transfection has been previously measured to be ∼5%.^41^ Additionally, reverse transcription inhibitor 3′-azido-3′-deoxythymidine (AZT; 4 μM, final concentration, Sigma, St. Louis, MO, USA) was added to culture media to block potential reinfection of the producer cells. p24 CA amounts were determined by using the HIV-1 p24 ELISA Kit (XpressBio, Frederick, MD, USA) according to the manufacturer’s instructions. The sensitivity of p24 detection was >1.7 pg/mL.

To test whether the P2A tag on A3G-D128K would affect A3G antiviral activity, pHL[WT] (3.3 μg) was co-transfected with pCMV-A3G(DK) or pCMV-A3G(DK)-P2A-eYFP (0.67 μg), and virus was harvested 48 h post-transfection. Normalized p24 CA amounts were used to transduce 4 × 10^3^ 293T cells in a 96-well plate, and luciferase activity was measured 48 h post-transduction using a 96-well luminometer (LUMIstar Galaxy, BMG LABTECH, Cary, NC, USA). Data were plotted as the percent inhibition of luciferase activity normalized to the HL[WT] control.

To determine the infectivity of A3G(DK), A3dr(DK), 3Gdr(DK), and A3x3G(DK) viruses, normalized p24 CA amounts were used to transduce target 293T cells (4 × 10^5^ cells per 6-well plate). Forty-eight hours post-transduction, the proportion of eYFP^+^ cells was analyzed by flow cytometry using the FACSCalibur system (BD Biosciences, San Jose, CA, USA) and FlowJo software (Ashland, OR, USA).

### Determination of Direct Repeat Deletion Frequency

To determine the frequency of A3G ∼900-bp direct repeat deletion and A3G-D128K gene reconstitution, virus produced from vector A3x3G(DK) was used to transduce 293T target cells and placed on puromycin selection. Genomic DNAs from independent cell pools were isolated (∼2 × 10^5^ puromycin-resistant cells per pool); and, using primers flanking A3G(DK), PCR amplification was performed to detect the proportion of undeleted A3x3G(DK) (2,190 bp) or deleted A3G(DK) (1,290 bp) proviruses. Primers CMVfor (5′-CAGAGCTCGTTTAGTGAACC-3′) and A3GendREV (5′-GATTCTGGAGAATGGCCCGC-3′) flank A3G(DK). In addition, individual puromycin-resistant 293T cell clones were isolated from cells transduced with virus from vector pA33G(DK) and analyzed to determine direct repeat deletion frequency by PCR amplification using primers CMVfor and GendREV4431 (5′-GGCTGTGCTCATCTAGTCCATC-3′) (undeleted, 1,864 bp; deleted, 964 bp).

### CEM or PM1 Cells Expressing A3G-D128K

CEM, PM1, or CEMSS cells (2 × 10^5^ cells per well in 96-well plate) were spin-infected with A3x3G(DK) virus (40 ng p24 CA) for 2 h, 1,200 × g, at room temperature in a Beckman Allegra 21R centrifuge (rotor S2096). Four days later, the cells were placed on puromycin selection (0.2 μg/mL; GIBCO) and allowed to expand with gradual increases in puromycin to a final concentration of 0.5 μg/mL. Flow cytometry was used to determine when >95% of the cells expressed eYFP. We sorted some cell pools first for eYFP^+^ cells using the BD FACSAria II system (BD Biosciences) before placing them on puromycin selection. CEM/D128K cell pool A consisted of ∼1.6 × 10^4^ independent transduced cells, whereas cell pools B, C, and D contained ∼1.4 × 10^5^ independent transduced cells. PM1/D128K cell pool A consisted of ∼800 independent transduced cells, whereas pools B and C ranged from 3,200 to 5,600 independent transduced cells. For isolation of CEMSS/D128K cell clones, puromycin-resistant cell pools were further diluted to single-cell clones and analyzed by western blot for expression of A3G-D128K.

### Cell Growth Assays

CEM/D128K, PM1/D128K, and CEMSS/D128K cell lines were plated at 2.5 × 10^5^ cells per well in 6-well plates in triplicate. The densities of viable cells were determined on days 2, 3, 4, and 5 by using a trypan blue exclusion assay (GIBCO) on a Cellometer Auto T4 cell counter (Nexelcom Biosciences, Lawrence, MA, USA).

### Western Blot Detection

Cell lystates were prepared using CelLytic M (Sigma) solution containing Protease Inhibitor Cocktail (Roche, Mannheim, Germany), followed by centrifugation at 10,000 × *g* for 10 min in a Heraeus Biofuge Fresco centrifuge to remove cellular debris. The cell lysates were mixed with NuPAGE LDS Sample Buffer (Invitrogen) containing β-mercaptoethanol and heated for 5 min at 95°C, and the samples were analyzed on 4%–20% Tris-glycine gels (Invitrogen) using standard western blotting techniques. For detection of A3G, the rabbit anti-A3G antiserum ApoC17 was used at a dilution of 1:5,000, obtained through the NIH AIDS Reagent Program, Division of AIDS, NIAID (from Dr. Klaus Strebel; catalog #10082).^92^^,^^93^ For detection of A3F, the rabbit polyclonal anti-human APOBEC3F (A3F) antibody (C18) was used at a dilution of 1:500, obtained through the NIH AIDS Reagent Program, Division of AIDS, NIAID (from Dr. Michael H. Malim; catalog #11474).^94^ For detection of α-tubulin, mouse anti-α-tubulin antibody (catalog #T9026, Sigma) was used at a 1:10,000 dilution. For detection of P2A, the rabbit anti-P2A antibody (catalog #ABS31, Sigma) was used at a 1:10,000 dilution. For detection of p24 CA, antibody against HIV-1 p24 (monoclonal, 1:10,000 dilution) was obtained through the NIH AIDS Reagent Program, Division of AIDS, NIAID: Anti-HIV-1 p24 Gag Monoclonal (#24-3) from Dr. Michael Malim (catalog #6458).^95^ An IRDye 800CW-labeled goat anti-rabbit secondary antibody (catalog #926-32211, LI-COR, Lincoln, NE, USA) was used at a 1:10,000 dilution to detect rabbit primary antibodies, and an IRDye 680-labeled goat anti-mouse secondary antibody (catalog #926-68070, LI-COR) was used at a 1:10,000 dilution to detect mouse primary antibodies. Protein bands were visualized and quantified using an Odyssey infrared imaging system (LI-COR).

### Virus Replication Assays

Parental CEM, CEMSS, and PM1 cells, as well as CEM/D128K, CEMSS/D128K, and PM1/D128K cells, were seeded at 1 × 10^6^ cells in 100 μL media and infected with NL4-3 virus (70 pg p24 CA) or equivalent virus from subtype AE, subtype C, HIV-2, SIV, or supernatants from multi-round infection experiments. For HIV-2 and SIVmac239, virus produced from transfected 293T cells that produced the same amount of luciferase activity upon infection of TZM-bl cells with 70 pg p24 CA-containing NL4-3 virus were used to infect the CEM/D128K and PM1/D128K cell lines. Note that SIVmac239 can use the alternate coreceptor BOB/GPR15 to infect the CEM cell line, which is CD4^+^/CXCR4^+^/CCR5^−^/BOB^+^.^96^^,^^97^ The virus-cell mixtures were incubated at 37°C and 5% CO_2_ for 5 h, and the cells were washed and placed in 3 mL media. At 2- or 3-day intervals, the virus-cell suspension was mixed, and 1 mL culture was removed and centrifuged at 16,000 × *g* for 10 min in a Heraeus Biofuge Fresco centrifuge. The resulting virus supernatant was removed and stored at −80°C, while the cell pellet was frozen and subsequently used for genomic DNA extractions. Of the remaining 2 mL culture, 1 mL culture was discarded, and the remaining 1 mL was resuspended with 2 mL fresh media. The sample was then incubated for another 2–3 days, and the process was repeated for the duration of the experiment. To assay for replication kinetics, the amount of HIV-1 virus in culture supernatants was determined by quantifying the amount of p24 CA by ELISA. The amounts of HIV-2 and SIVmac239 virus in culture supernatants were determined by infection of TZM-bl cells and quantifying the luciferase activity 48–72 h after infection.

### Viral DNA Isolation, Sequencing, and Hypermutation Analysis

Total genomic DNA from transduced or infected cells was isolated using the QIAamp DNA Blood Mini Kit (QIAGEN, Hilden, Germany), and an 896-nt Vif region from integrated proviruses was amplified by PCR using primers NL43-seq-4921F (5′-GAGATCCAGTTTGGAAAGGAC-3′) and NL43-seq-5816R (5′−CTGTCGAGTAACGCCTATTC-3′). Alternatively, a 982-nt *puro*-WPRE (woodchuck hepatitis virus post-transcriptional regulatory element) region was amplified using primers puroFor (5′-CGCGCAGCAACAGATGGAAG-3′) and nefRev (5′-CTTGTGCTTCTAGCCAGGCA-3′). The PCR products were resolved on a 1% agarose gel, the 896-bp band was extracted from the gel using the QIAquick Gel Extraction Kit (QIAGEN), and the DNA was cloned into a TOPO TA plasmid (Invitrogen). The plasmid DNA was extracted from the bacterial colonies using the NucleoSpin 8 Plasmid Kit (Clontech). Individual clones were sequenced (Macrogen) and evaluated for nucleotide and amino acid changes and further analyzed for the presence of hypermutation using Hypermut (https://www.hiv.lanl.gov/content/sequence/HYPERMUT/hypermut.html). Sequenced clones containing single G-to-A changes were not considered hypermutated, as single mutations may have resulted from errors during PCR or sequencing.

### Infection of CD4^+^ T Cells and CD34^+^ HSPCs

PBMCs were grown in RPMI medium supplemented with 10% fetal calf serum (HyClone) and 1% penicillin-streptomycin (final concentration: penicillin, 50 U/mL; and streptomycin, 50 μg/mL; GIBCO). PBMCs were stimulated for 3 days at 37°C with IL-2 (40 U/mL; Sigma) and PHA-P (5 μg/mL; Sigma) or Dynabeads Human T-activator CD3/CD28 (Invitrogen). Cells were washed to remove PHA-P, and CD4^+^ T cells were isolated using the Dynal CD4 Positive Isolation Kit (Invitrogen). Flow cytometry analysis was performed to determine the percent activated CD4^+^ T cells in the isolated population using the following antibodies (BD PharMingen, San Jose, CA, USA): fluorescein-isothiocyanate (FITC)-conjugated mouse anti-human CD4, FITC-conjugated mouse immunoglobulin (Ig)G κ isotype control, phycoerythrin (PE)-conjugated mouse anti-human CD25, and PE-conjugated mouse IgG1 κ isotype control. Activated CD4^+^ T cells were spin-inoculated with virus at an MOI of 10 (1,200 × g, 1 h, room temperature) in the presence of 4 μg/mL polybrene. Cell/virus mix was incubated for 20 h at 37°C. Subsequently, the cells were washed, and flow cytometry analysis was performed 72 h post-transduction to determine the proportion of eYFP^+^ cells. Culturing and infection of CD34^+^ HSPCs were performed as previously described.^98^

Umbilical cord blood CD34^+^ HSPCs were pre-stimulated for 24 h in Iscove’s modified Dulbecco’s medium (IMDM) supplemented with 20% BIT 9500 (STEMCELL Technologies, Cambridge, MA, USA), 50 ng/mL each of human interleukin-6 and thrombopoietin, 150 ng/mL human stem cell factor, and 100 ng/mL Flt-3 ligand (PeproTech, Rocky Hills, NJ, USA). After pre-stimulation, 2 × 10^4^ cells per well were transduced with the LV-GFP or A3x3G(DK) lentiviral vector at an MOI of 20 in the presence of 5 μg/mL polybrene for 24 h.^98^ After lentiviral vector transduction, HSPCs were washed to remove vector, cultured for 6 days, and then analyzed by flow cytometry for the proportion of GFP^+^ or eYFP^+^ cells, as previously described.^98^

## Author Contributions

V.K.P., K.A.D.-F., and W.-S.H. conceptualized the project and designed experiments. K.A.D.-F. and N.D.T. performed experiments. D.A., O.A.N., and M.H. provided technical support. V.K.P. and K.A.D.-F. wrote the manuscript, and all authors reviewed and edited the manuscript.

## Conflicts of Interest

The authors declare no competing interests.

## Supplemental Information

Document S1. Figures S1–S4

Document S2. Article plus Supplemental Information
