# Supplementary material for: Development of Lentiviral Vectors for HIV-1 Gene Therapy with Vif-Resistant APOBEC3G
Source: Mol Ther Nucleic Acids. 2019 Oct 31;18:1023–38. doi: 10.1016/j.omtn.2019.10.024 (PMC6889484; doi:10.1016/j.omtn.2019.10.024)
Supplement: Document S1. Figures S1–S4 [file mmc1.pdf]

OMTN, Volume *18*

Supplemental Information

Development of Lentiviral Vectors for HIV-1 Gene Therapy with Vif-Resistant *APOBEC3G*

Krista A. Delviks-Frankenberry, Daniel Ackerman, Nina D. Timberlake, Maria Hamscher, Olga A. Nikolaitchik, Wei-Shau Hu, Bruce E. Torbett, and Vinay K. Pathak

Supplemental Figure S1

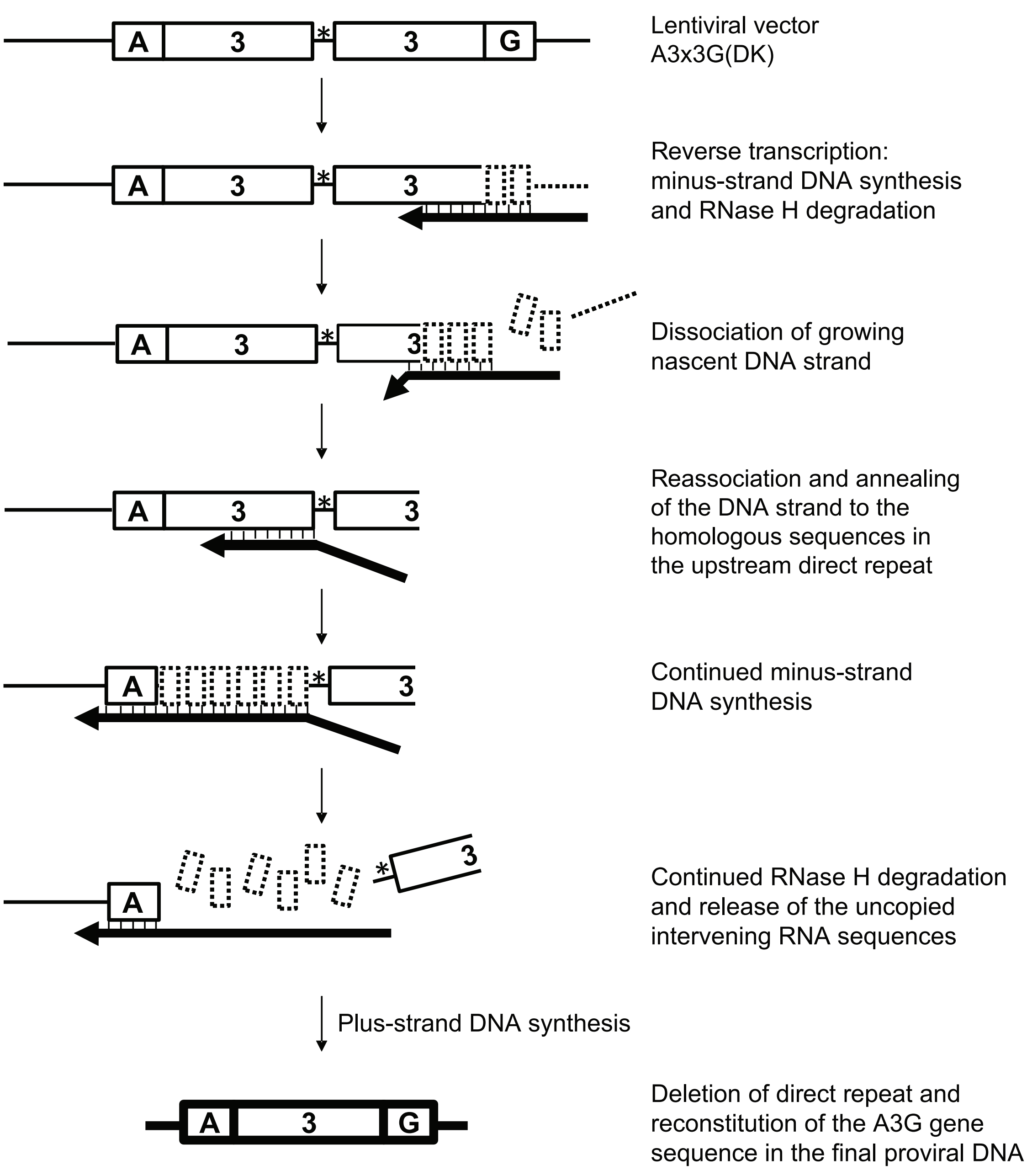

Figure S1. Dynamic Copy-Choice Model. Shown are the two ~900-bp direct repeats of A3G (white boxes labeled "3") undergoing an intramolecular template switch during minus-strand DNA synthesis (arrow and thick black line). Degradation of the RNA template (dotted lines) allows the newly synthesized DNA strand to dissociate and anneal to the acceptor template (upstream "3" direct repeat). Reverse transcription continues resulting in deletion of one of the directly repeated sequences (plus the intervening stop codons, shown as "*") and reconstitution of the A3G gene sequence.

Supplemental Figure S2

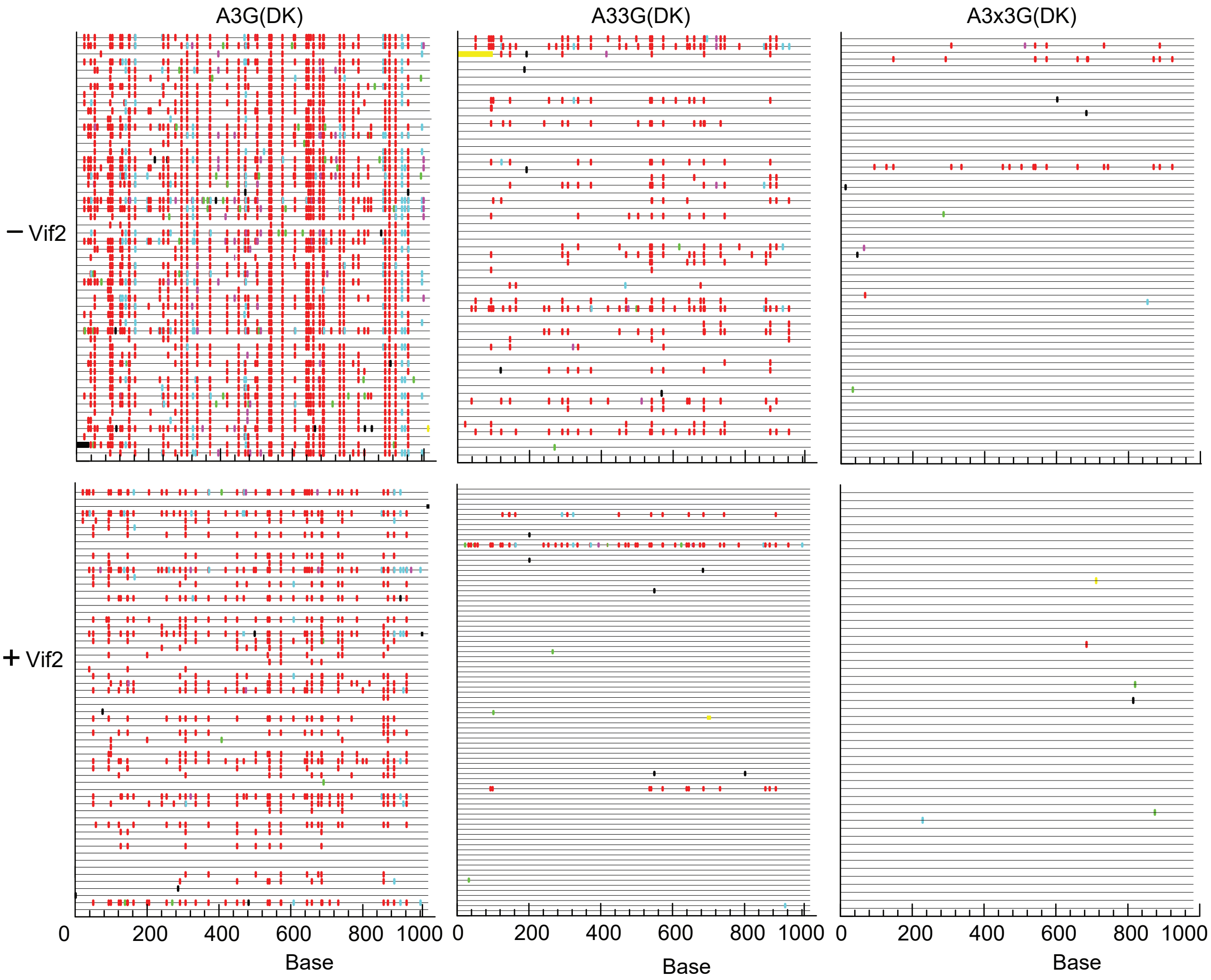

Figure S2. Vector A3x3G(DK) exhibits little to no G-to-A hypermutation in transduced target cells. Proviral clones from transduced 293T cells with vector virus A3G(DK), A33G(DK) and A3x3G(DK) produced in the presence or absence of Vif2 were analyzed for G-to-A hypermutation. Sequence analysis of a 982-bp region (end of *puro* to start of *nef*) is represented as Hypermut plots (http://www.hiv.lanl.gov/content/sequence/HYPERMUT/hypermut.html); colored vertical hash marks represent nucleotide differences from NL4-3 as defined by Hypermut (red = GG>GA, cyan = GA>AA, green = GC>AC, magenta = GT>AT, black = not G>A transition, gaps = yellow).

Supplemental Figure S3

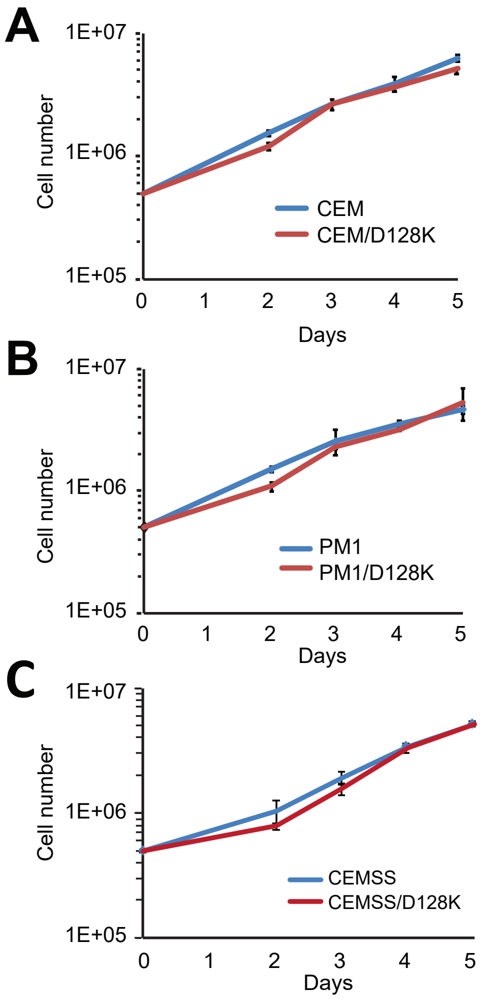

Figure S3. Growth kinetics of T cell lines expressing A3G-D128K. Cell growth kinetics were measured by cell counts over a five-day period for (A) CEM versus CEM/D128K, (B) PM1 versus PM1/D128K, and (C) CEMSS versus CEMSS/D128K cell lines. n= 3; error bars, strandard deviation.

Supplemental Figure S4

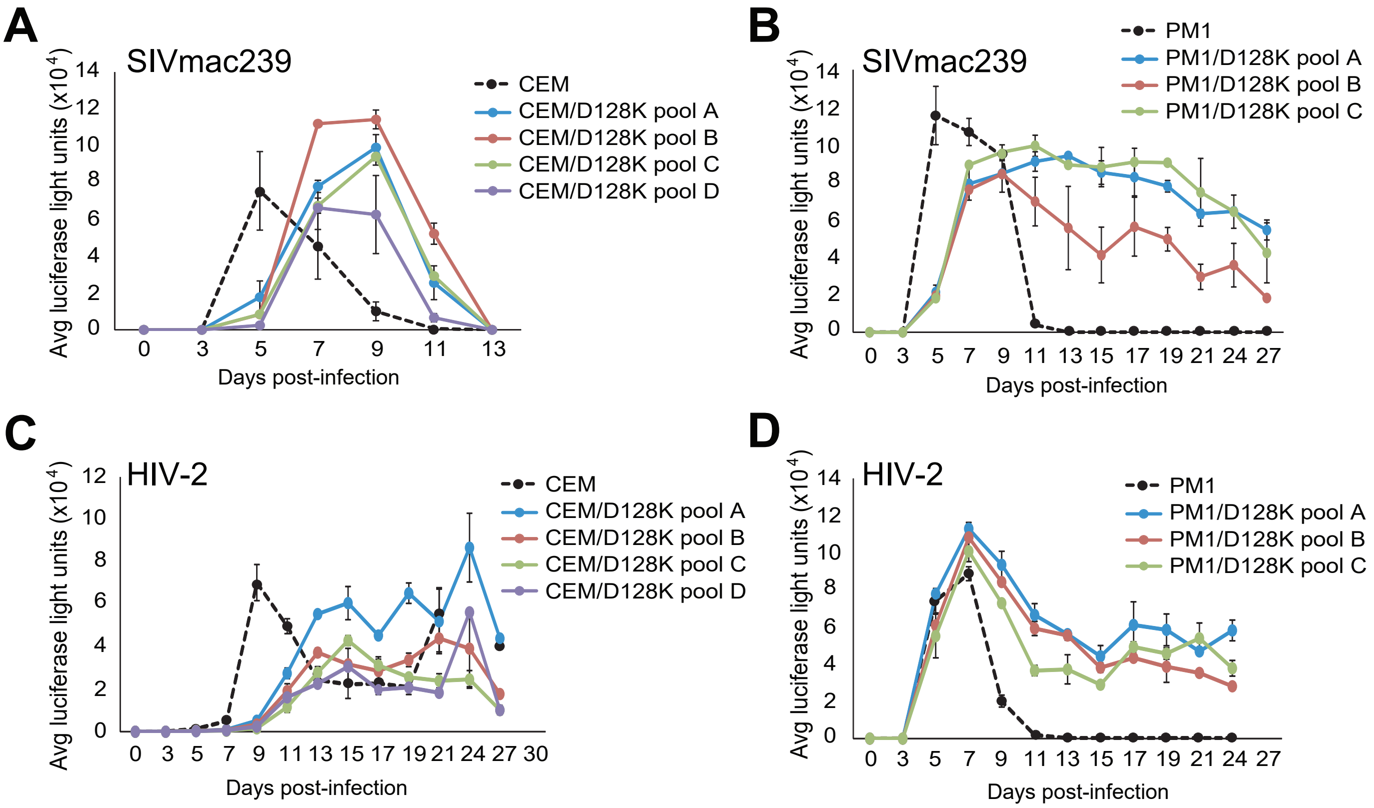

Figure S4. Replication kinetics of HIV-2 and SIVmac239 in CEM/D128K and PM1/D128K cell lines. Normalized virus from replication competent SIVmac239 (A and B) and HIV-2 (C and D) were used to infect four independent CEM/D128K or three PM1/D128K independent cell pools (2 flasks each). Supernatants were harvested every two to three days for 13 to 27 days, and viral production was assayed by infection of TZM-bl cells. Average luciferase light units 48 h post infection is shown for each infection over time. Error bars, standard deviation.
